# FOLFOX Therapy Induces Feedback Upregulation of CD44v6 through YB-1 to Maintain Stemness in Colon Initiating Cells

**DOI:** 10.3390/ijms22020753

**Published:** 2021-01-13

**Authors:** Shibnath Ghatak, Vincent C. Hascall, Roger R. Markwald, Suniti Misra

**Affiliations:** 1 Department of Regenerative Medicine and Cell Biology, Medical University of South Carolina, Charleston, SC 29425, USA or ghatak@musc.edu (S.G.); markwald@musc.edu (R.R.M.); 2 Hollings Cancer Center, Department of Biochemistry and Molecular Biology, Medical University of South Carolina, 86 Jonathan Lucas St, Charleston, SC 29425, USA; 3 Department of Biomedical Engineering/ND20, Cleveland Clinic, Cleveland, OH 44195, USA; hascalv@ccf.org

**Keywords:** CD44v6, YB-1, MDR1, CIC, stemness genes, CD44v6 CRISPR/Cas9 knockout, YB-1 CRISPR/Cas9 knockout, CD44v6-therapy, colorectal cancer (CRC)

## Abstract

Cancer initiating cells (CICs) drive tumor formation and drug-resistance, but how they develop drug-resistance characteristics is not well understood. In this study, we demonstrate that chemotherapeutic agent FOLFOX, commonly used for drug-resistant/metastatic colorectal cancer (CRC) treatment, induces overexpression of CD44v6, MDR1, and oncogenic transcription/translation factor Y-box-binding protein-1 (YB-1). Our study revealed that CD44v6, a receptor for hyaluronan, increased the YB-1 expression through PGE2/EP1-mTOR pathway. Deleting CD44v6, and YB-1 by the CRISPR/Cas9 system attenuates the in vitro and in vivo tumor growth of CICs from FOLFOX resistant cells. The results of DNA:CD44v6 immunoprecipitated complexes by ChIP (chromatin-immunoprecipitation) assay showed that CD44v6 maintained the stemness traits by promoting several antiapoptotic and stemness genes, including *cyclin-D1,* *BCL2,* *FZD1,* *GINS-1*, and *MMP9.* Further, computer-based analysis of the clones obtained from the DNA:CD44v6 complex revealed the presence of various consensus binding sites for core stemness-associated transcription factors “CTOS” (c-Myc, TWIST1, OCT4, and SOX2). Simultaneous expressions of CD44v6 and CTOS in CD44v6 knockout CICs reverted differentiated CD44v6-knockout CICs into CICs. Finally, this study for the first time describes a positive feedback loop that couples YB-1 induction and CD44 alternative splicing to sustain the MDR1 and CD44v6 expressions, and CD44v6 is required for the reversion of differentiated tumor cells into CICs.

## 1. Introduction

Colorectal cancer (CRC) is the second leading cause of cancer-related deaths in Western countries including the USA, with incidences increasing by 2% annually and has a dismal prognosis with a 14% 5-year survival rate of patients with metastasis [1,2]. Recent developments in cancer prevention endeavors, including the extensive application of colonoscopy and the identification and removal of precancerous lesions, have resulted in a considerable overall reduction in CRC incidence [3,4,5]. Conventional treatment for CRC includes the multicomponent drug FOLFOX that is composed of 5-fluorouracil (5-FU), oxaliplatin (OXA), and leucovorin. FOLFOX chemotherapy is widely used as a first-line chemotherapeutic agent which often fails because the targeted cancer cells acquire chemoresistance over time [6,7,8]. Even though the prognosis response rate to OXA is approximately 24%, acquired resistance progresses in nearly all patients after repeated treatment with either OXA alone, or with FOLFOX, eventually limiting its therapeutic efficacy [6,9]. Improved understanding of the mechanisms that contribute to acquisition of FOLFOX resistance by CRC cells is imperative for developing novel therapies to improve FOLFOX efficacy.

An important step in understanding mechanisms of cancer drug resistance in recent years, has been a growing body of evidence that tumor tissue is composed of heterogeneous, hierarchy of cells that differ in morphology, gene expression, proliferative capacity, and invasiveness [10]. This heterogeneity originates from a small subset of cancer cells, called cancer stem cells (CSCs) or cancer initiating cells (CICs), that are the unique source of all tumor cells and responsible for tumor propagation and relapse [11]. Interactions between tumor cells and their microenvironment create niches that promote CIC survival. Moreover, when this niche is disrupted, CICs initiate a series of cellular processes for self-renewal, replication, and differentiation in an effort to restore the tumor mass and its microenvironment [12,13,14]. Since the first identification of CSCs/CICs in breast cancer used a CD44/CD24 marker [15], CICs have been identified in a variety of solid tumors [16,17,18,19,20,21,22,23,24], including colon carcinomas [25,26,27]. Unlike naturally occurring somatic stem cells, CICs initiate tumorigenic activity when transplanted into animals [28,29]. Moreover, variation in the genetics and epigenetic damages of CRC patients is so different that markers to detect CICs from more differentiated progeny have not been completely informative across all patient tumors [30,31,32]. In addition, most CIC enhancement markers mediate interactions between a tumor cell and its stromal environment, indicating that the tumorigenic characteristics associated with that marker may be lost after depletion of CICs from their microenvironment. Similarly, human pancreatic CICs expressing the marker CD133 and the chemokine SDF-1 receptor CXCR4 lost their metastatic activity when the CXCR4^+^/CIC population was depleted. This implies that CICs may take over information associated with the marker (in this case, SDF-1/CXCR4-controlled pathways) to initiate metastasis [33]. CRC cells expressing CD166 [34], CD44 [35], CD44v6 [27], CD66c [34], CD133 [36], and aldehyde dehydrogenase (ALDH1) [37] describe CIC characteristics. However, the cell-surface markers that recognize CICs and have a functional role in the antiapoptotic signaling to drive tumorigenesis have remained poorly defined.

Our working hypothesis for this study is that CICs are involved in the development of drug-resistance, treatment failure, and tumor relapse in cancer [38,39]. We base this hypothesis on observations that CICs acquire high levels of antiapoptotic proteins, possess low levels of reactive oxygen species, and demonstrate an enhanced efficiency of DNA damage repair [36,40,41]. Irrespective of the marker used, CICs are the parental source for which all other malignant cells within a given tumor arise and are responsible for CRC tumor growth/maintenance, metastatic spread, resistance to conventional chemotherapies, and relapse after cancer therapy [11,42]. Therefore, CRC CICs must be effectively targeted to inhibit tumor growth and improve survival of patients with CRC. Our findings indicate that one mechanism for targeting them is to modulate the expression of a splice variant of CD44.

CD44 is a multistructural and multifunctional transmembrane glycoprotein that acts as a receptor for hyaluronan (also called hyaluronic acid). CD44 is encoded by a single gene containing 20 exons, 10 of which are alternatively spliced to generate the numerous CD44 splice variants (CD44v) [43,44]. The standard isoform of CD44 (CD44s) is small with no variant exons and is nearly ubiquitous on vertebrate cells [45]. Variant 6 of CD44 (CD44v6) participates in tumor development and progression in many ways that are restricted to stem cell subpopulations [27,46]. In agreement with this, CD44v6, but no other variants, promotes generation of gut adenomas (tumors) in mouse models of familial adenomatous polyposis [47,48]. Its role in CRC progression derives from its ability to bind ligands associated with both tyrosine kinase receptors or non-tyrosine kinase receptors including c-Met, VEGF, TGFβ1, and ERB2 [48,49,50,51,52,53,54,55,56,57,58], leading to changes in biological activities such as activation of antiapoptotic signaling, cell-matrix adhesion, cell migration, proliferation, differentiation, and survival [59,60]. Much progress has been made in the assessment of the molecular structures and functions of the standard isoform (CD44s) and of its various isoforms in cancer cell signaling in general rather than by analysis of CIC signaling. Recent studies indicate that CD44 variants are restricted to CIC populations and promote tumor development in animals [61]. CD44v6 positive (+)/CICs have been associated with increased metastatic behavior in both pancreatic cancer [33,44,62,63] and CRC [27,46], suggesting that CICs may takeover CD44v6-regulated apoptosis resistance-signaling pathways to initiate drug-resistance. Experiments using knock-in mice that express either CD44v4-10 or CD44s isoforms have demonstrated that CD44v isoforms, but not the CD44s isoform, promote adenoma formation in Apc (Min/+) mice [47]. Thus, CD44v6 predicts poor prognosis and is a marker of constitutive and reprogrammed CICs that drive CRC metastasis [27].

Although CICs and somatic stem cells exhibit similar transcription factors (TFs), including SOX2, OCT4, NANOG, KLF4, and c-Myc, several studies have demonstrated that the abnormal expression of some distinctive TFs [64] have a crucial role in the reprogramming of these cells [65]. In this case, overexpression of TFs results in dysregulation of associated signaling pathways that are linked with CIC lineage and differentiation phenotype [66]. Transcriptional regulation occurs when certain TFs bind to the DNA at binding sites of a promoter and affect the transcription of the regulated gene via interactions with their gene promoters [65,66]. In this circumstance, TFs may have a crucial role in the maintenance of CIC stemness. Transcription factor networks can be involved in gene regulatory networks [67,68], and dictate cell phenotypes when expressed in various settings in an ectopic manner [69,70,71]. Several core transcription factors, including TWIST1, Snail, Zeb 1 [72,73] as well as OCT3/4 [74,75], SOX2 [76], and NANOG [77,78] have pivotal roles in maintenance of pluripotency in both early embryos and embryonic stem cells, adult stem cells, and CICs. In addition, induced pluripotent stem cells (iPSC) can be directly generated from fibroblast cultures by the addition of some of these core TFs [79]. Besides this, several genes that are frequently upregulated in tumors, such as STAT3 [80,81] and β-catenin [82,83], have been shown to contribute to the long-term maintenance of the stem cell traits. CRC CICs exhibit characteristics comparable to normal stem cells that could be associated with the expression of similar TFs including SOX2, OCT4, NANOG, KLF4, Lgr5, TWIST1, and c-Myc, and signaling pathways including WNT/β-catenin pathways directed for CRC propagation [65,84,85]. Many studies indicate that the Y-box-binding protein-1 (YB-1) transcription factor can function as an oncoprotein [86,87,88] to regulate stemness, drug-resistance and tumorigenic properties in various cancers [89,90,91,92,93,94] including CRC [95]. As mentioned above, CD44v6, a marker of CICs, drives CRC metastasis [27]. A recent study shows that YB-1 binds to the CD44 promoter to transcriptionally upregulate its expression in breast cancer [96]. PGE2 was shown to induce YB-1 expression, which is involved in the drug resistance and malignance of several carcinomas [97,98,99]. While we and others have shown that CD44 regulates MDR1 expression in various cancers [49,100,101] and controls COX2-PGE2 signaling in CRC [50,102], whether YB-1 may be linked to the ability of CD44v6 to induce the expression of genes linked to stemness and drug resistance in CD44v6+CICs is not known. Therefore, this study was designed to assess our hypothesis that FOFOX-induced CD44v6/COX2-PGE2/mTOR may promote CRC resistance through upregulation of YB-1 signaling that promotes CD44v6 splicing, and that CD44v6 then sustains YB-1 signaling. The novelty of our results in this study is that it provides the first demonstration of a positive feedback loop linking signaling-dependent alternative splicing of CD44 to the drug-resistance gene MDR1 through YB-1. Further, our results revealed for the first time that CD44v6 regulated YB-1 signaling is required for the maintenance of FOLFOX resistance and for the reversion of differentiated CD44v6 knockout-CICs into CICs with stemness traits.

## 2. Results

### 2.1. Upregulation of CD44v6 and YB-1 Contributes to Acquired Chemoresistance and Stemness in Colon Cancer SW948 Cells

To determine the mechanism of FOLFOX resistance in CRC, we developed a cellular model of FOLFOX resistance. We screened seven CRC cell lines for *CD44v6* expression and selected few of them including SW948 cells that exhibited lower steady-state expression of CD44v6 (Supplemental Figure S1A). In order to determine the mechanism of FOLFOX (combination of 5-fluorouracil (5-FU) + Oxaliplatin (OXA) + leucovorin) resistance in CRC cells, we determined the IC_50_ values of 5-FU and OXA for inhibiting SW948 CRC cell growth using a cell viability assay (assessed by ATP based assay (Cell Titer-Glo)) in the presence of increasing concentrations of these chemotherapeutic drugs. The average IC_50_ value for 5-FU of SW948 cell is ≈60 µg/mL, and the average IC_50_ value for OXA in these cells is ≈5–10 µg/mL (Supplemental Figure S1B,C). The average IC_50_ value for FOLFOX is shown in Supplemental Figure S1D. Next, we evaluated the kinetics of CD44v6 induction upon exposure to 1 × FOLFOX (1x FOLFOX = IC_50_ of 5-FU + IC_50_ OXA + 1 µM leucovorin). Resistance from either 5-Fluorouracil (5-FU) or Oxaliplatin (OXA), two components of FOLFOX, has been associated with increased CD44v6 mRNA expression in CRC cells [103].Thus, in order to determine whether FOLFOX resistance is associated with CD44v6, serum depleted SW948-S cells were stimulated by addition of 1 × FOLFOX in media.

**Figure 1:**
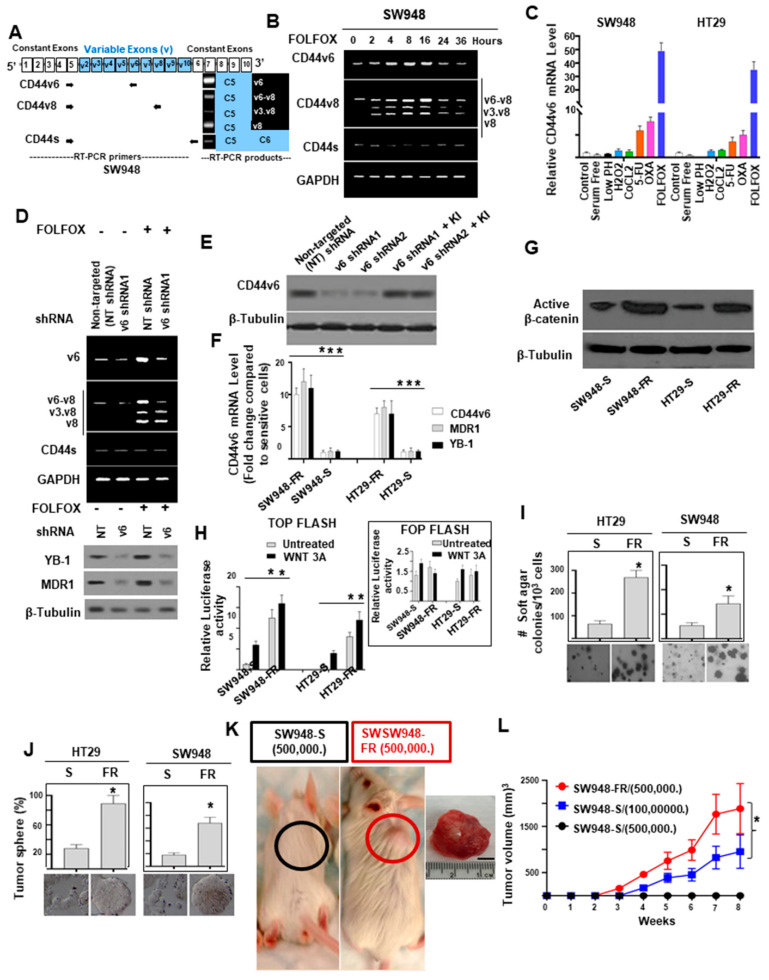
Establishment of FOLFOX resistant colorectal cancer (CRC) cells that exhibit increased CD44v6 expression and signaling. (**A**) A schematic diagram of the CD44 gene, where constitutive (c) and variable (v) exons, and the PCR primers used to amplify CD44 variable (v) and standard (s) isoforms are shown. The primers for both the CD44v6 and CD44s predominantly generate one PCR product, whereas the primers for the CD44v8 variants amplify three variant PCR products. (**B**) A time course of FOLFOX (FOLFOX: 50 μg/mL 5-Flurouracil + 10 μM Oxaliplatin + 1 μM leucovorin) stimulation on CD44 isoform mRNA expressions (analyzed by semiquantitative RT-PCR) in SW948 cells was depicted. (**C**) QPCR assays for variant 6 of CD44 (CD44v6) expression under low-pH (ischemic stress), CoCl2 (hypoxic stress), H2O2 (oxidative stress), 5-FU, OXA, and FOLFOX treatment (chemotherapeutic stress) in SW948 cells are shown. (**D**) shRNA-mediated knockdown of CD44v6 affects alternative splicing of CD44 and downregulates YB1 and MDR1 expression. (**E**) Validations of CD44v6 shRNAs were done by the indicated shRNA mediated knockdown and the corresponding shRNA resistant knock-in (KI) gene overexpression. (**F**) SW948-FR and HT29-FR cells selectively overexpressed CD44v6, MDR1, and YB-1 mRNAs (by QPCR) compared to sensitive (“S”) pairs of cells. The expression of indicated proteins in FR cells compared to sensitive pairs are presented as mean ± SD (*n* = 3); *, *p* < 0.01. Student’s *t* test was used to assess the significance. The experiment was performed three times and representative data are shown. (**G**) Western blot (WB) analyses for β-catenin and β-tubulin of “S” and “FR” cell lysates of SW948 and HT29 cells are shown. (**H**) β-catenin luciferase activity of “S” and “FR” lysates of SW948 and HT29 cells treated with or without WNT 3A are shown. The relative luciferase in FR cells compared to sensitive pairs are presented as mean ± SD (*n* = 3); *, *p* < 0.01. Student’s *t* test was used to assess the significance. The experiment was performed three times and representative data are shown. (**I**) Anchorage-independent growth in soft agar is shown for SW948-FR and HT29-FR cells and compared with their “S” pairs (magnification, 50 ×). (**J**) Tumor-sphere formation assay was done for the SW948-FR and HT29-FR cells and compared with their “S” pairs (magnification, 100×). (**K**,**L**) Tumor formation is shown in nude mice injected with 500,000 SW948-FR cells or with 500,000 SW948-S cells. SW948-FR cells formed tumor nodules in all injected mice (8/8), whereas SW948-S cells did not induce any tumor nodules until 5 months (left, 0/7 mice) (**K**). Growth curves are shown for these xenograft tumors in BALB/c nude mice (**L**). Values in C, I, and J represent means ± SD; *n* = 3–6; * *p* < 0.05 for FOLFOX resistant cells compared to sensitive cells. Scale bar, 50 μm.

We first examined the expression profile of CD44 variants in SW948 cells after stimulation with FOLFOX by exon-specific reverse transcription-PCR (RT-PCR) analysis (Figure 1A). Several variant isoforms are indeed expressed. Exon v6 seems to be expressed together with exons *v6–v8* and also as an independent isoform (shown in Figure 1A). The expression levels of CD44 variants were examined by RT-PCR using different sets of primers (Figure 1A). The variants were detected using a 5′ primer from a constitutive exon 5 of CD44 and two distinct 3′-primers complementing to v6, and v8 exons of CD44, respectively. In addition, the CD44s standard form having no alternate splicing was detected using primers for the constitutive exons 5 and 6 of CD44. The CD44v6 primers and CD44s primers each principally amplified a single product (Figure 1A). The v8 primer gave rise to three alternately spliced variants of CD44 containing (1) variant exons v6, v7, and v8 (illustrated as v6–v8); (2), variant exons v3 and v8 (illustrated as v3.v8); (3) and variant exon v8 (shown as v8), all joined to the 5′-constitutive exon 5 (Figure 1A). All products were confirmed by DNA sequencing as described [58]. Following 24 h of serum starvation, the relative expressions of CD44 variants were low, while stimulation with FOLFOX upregulated v6 mRNA expression that peaked between 4 and 16 h and returned to basal levels at 24–48 h likely due to the exhaustion of FOLFOX within the media (Figure 1B; primers are in Supplemental Table S1).

To explore whether the induction of CD44v6 expression by FOLFOX was not modulated by various stress environments such as ischemic, or hypoxic, or oxidative stress conditions, we examined the expression of CD44v6 in SW948 cells by treating them with various chemical agents for 48 h. To create ischemic, and/or oxidative stress conditions, we cultured the cells in serum-free medium. For hypoxic stress we employed low-pH condition, 300 μM CoCl2 and for creating oxidative stress, we used 300 μM H_2_O_2_. In addition, for chemotherapeutics induced cytotoxic stress, we used 60 μM 5-FU, or 5–10 μM OXA (IC_50_ of 5-FU (Supplemental Figure S1B), and IC_50_ of OXA for SW948 cells (Supplemental Figure S1C), respectively) as well as 1 × FOLFOX in culture medium. CD44v6 expressions were determined using QPCR analysis. Our data demonstrated that basal CD44v6 expression was very low in SW948 cells but significantly increased with chemotherapeutics (5-FU, or OXA, or FOLFOX), whereas stress creating chemical agents did not induce CD44v6 expression (Figure 1C). Similar results were found in HT29 cells (Figure 1C). Overall, our data indicate that in CRC cells (SW948 and HT29 cells), FOLFOX and its components 5-FU and OXA considerably and distinctively induced CD44v6 expression, which could interact with various cellular targets and offer one of the fundamental mechanisms for the drug resistance in CRC cells.

Resistance to chemotherapeutics has been independently associated with increased CD44v6 expression [26,27], and YB-1 overexpression has already been reported to be associated with possible chemoresistance through the regulation of MDR1 in breast cancer [99] and multiple myeloma cells [104]. Therefore, we postulated that CD44v6 and YB-1 may be associated to FOLFOX resistance in SW948 cells. To address this, we evaluated the effects of FOLFOX on protein expressions of YB-1 and the ATP-binding cassette subfamily B member 1 (ABCB1), also known as multidrug-resistance-1 (MDR1), protein expressions in SW948 cells following treatment with or without FOLFOX and CD44v6 shRNA. Knockdown of CD44v6 in SW948 cells downregulated both YB-1 and MDR1 protein expressions and inhibited the FOLFOX-induction of v6-containing variants but not CD44 standard (CD44s), v3.v8 or v8 variants (Figure 1D). Validations of CD44v6 shRNA1 and CD44v6 shRNA2 are shown in Figure 1E. Overall, these results indicate that CD44v6 has key roles for YB-1 and MDR1 expressions in response to FOLFOX.

To further determine the mechanism of FOLFOX resistance, we used serially escalated doses of FOLFOX (1 × FOLFOX–5 × FOLFOX) in parent sensitive CRC cells (SW948-S and HT29-S) to generate FOLFOX-resistant (FR) cells. After recovering from 1 × FOFOX, cells were treated with 2 × FOLFOX. The survived cells were then treated with 4 × FOLFOX to delete most of the cell population. After high-dose 5 × FOLFOX treatment, a small number of cells survived and slowly repopulated to form colonies. Finally, FOLFOX-resistant CRC cells were established, and the IC_50_ values of FOLFOX in SW948-FR and HT29-FR cells were 3.5 × FOLFOX and 2.8 × FOLFOX (*p* < 0.001, compared with each parental cell). The response of the SW948-FR and HT29-FR cells to FOLFOX treatment resistance were evaluated using QPCR assay for CD44v6, YB-1 and MDR1 mRNA expressions and compared to their sensitive pairs (Figure 1F). SW948-FR and HT29-FR cells have significantly higher levels of CD44v6, YB-1, and MDR1 expressions compared to their sensitive pairs SW948-S and HT29-S (Figure 1F).

Given that activation of the WNT/β-catenin pathway is the hallmark of colorectal cancer initiating cells (CRC-CICs) [27] and because CICs are naturally resistant to chemotherapy through their quiescence, capacity for DNA repair, and ABC transporter expression [105], we evaluated stemness in FR cells. Figure 1G,H shows that FOLFOX promoted stemness in FR cells via a WNT/β-catenin pathway with higher active β-catenin expression and increased β-catenin/TCF4 TOP-Flash transcriptional activity. To determine the clonogenicity of FR cells in vitro, we compared their clonal capacity to sensitive pairs employing a soft agar colony formation assay. Compared to parental sensitive cells, FR cells were able to form increased anchorage-independent growth assessed by formation of soft agar colonies (Figure 1I). It has been recently documented that CRC-CICs could be expanded as tumor-spheres [26,27]. Therefore, we investigated sphere-forming activity of both parental and FR cells. Compared with parental sensitive cells, FR cells were able to form significantly greater numbers of tumor-spheres in serum free medium (Figure 1J). Next, to evaluate, whether the effects of FOLFOX resistance offer increased in vivo tumor growth compared to that implanted with the corresponding sensitive cells, we implanted 5 × 10^5^ cells of sensitive and resistant FR cells of SW948 into immunocompromised mice (# of mice = 7 for sensitive and # of mice = 8 for FR cells implanted xenograft tumor for each of the experiments; *n* = 3 experiments). In agreement with the soft agar growth and tumor sphere formation results (Figure 1I,J), 5 × 10^5^ resistant cells generated tumors in at least 90–100% of immunocompromised mice injected from SW948-FR cells (Figure 1K, red, tumor formation = 8/8 mice), whereas 5 × 10^5^ sensitive cells (SW948-S) were not adequate to form tumors (Figure 1K, black, tumor formation = 0/7 mice). However, implantation of 20-fold more sensitive SW948-S cells (1 × 10^7^) were able to initiate tumors in three independent experiments. When tumor volumes were examined every day to evaluate the latency, tumors initiated from 5 × 10^5^ SW948-FR cells began to increase at 2 weeks while tumors initiated from 1 × 10^7^ SW948-S cells began to increase later at 3–4 weeks and had much smaller size at 8 weeks (900 mm^3^ compared to 1800 mm^3^ at 8 weeks, Figure 1L). The results from Figure 1G–L provide evidence that FOLFOX-resistant FR cells were more tumorigenic in vitro and in vivo and had greater sphere-forming activity than parental sensitive cells, which are hallmark characteristics of CRC-CICs. This suggests that expansion of CICs might have an important role for the acquisition of FOLFOX resistance.

### 2.2. Expansion of CD44v6 (+) CICs during Acquisition of FOLFOX Resistance

**Figure 2:**
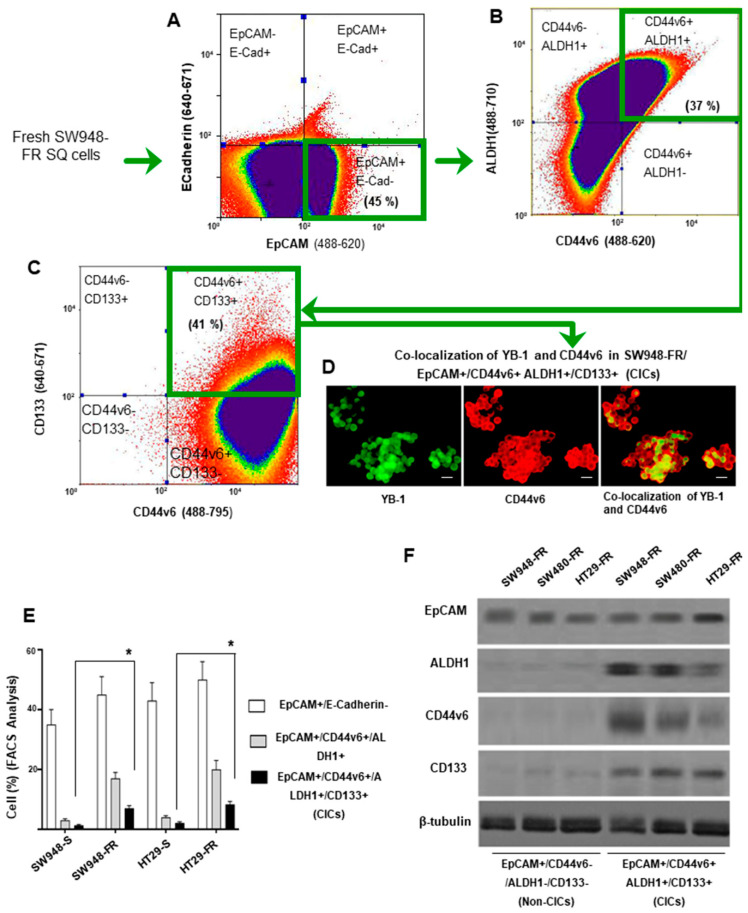
Flow cytometric analyses of EpCAM+/CD44v6+/ALDH+/CD133+ cells in SW948-FR cells isolated from SW948-FR/subcutaneous (SQ) tumors. Alexa fluor-tagged antibodies at the indicated laser lines were used to isolate: (**A**) EpCAM+/ECadherin- cells; (**B**) CD44v6+/ALDH1+ cells from EpCAM+/ECadherin-cells; (**C**) CD133+ cells from CD44v6+/ALDH1+ cells. (**D**) Immunofluorescence staining shows colocalization of CD44v6 (Red) and YB-1 (Green) in EpCAM+/CD44v6+/ALDH1+/CD133+ (CICs), scale bar, 50 μm. (**E**) Percentages of EpCAM+/E-cadherin-, CD44v6+/ALDH1+, and CD44v6+/ALDH1+/CD133+ on sorted cells were assessed by flow cytometry on freshly purified CRC cells isolated from subcutaneous (SQ) SW948-FR tumor cells. (**F**) Western blots are shown for EpCAM+/CD44v6-/ALDH-/CD133- (Non-CICs) and EpCAM+/CD44v6+/ALDH+/CD133+ (CICs) by probing with E-Cadherin, EpCAM, CD44v6, ALDH1, YB-1, and CD133 antibodies. FACS, immunofluorescence and WB data are representative of three experiments. Enrichment of CICs in FR cells compared to sensitive pairs in Figure 2E are presented as mean ± SD (*n* = 3); *, *p* < 0.01. Student’s *t* test was used to assess the significance. The experiment was performed three times and representative data are shown.

Given that FOLFOX maintained the stemness feature of CRC cells (Figure 1F–K), CICs were isolated from the freshly dissociated subcutaneous tumors (SQ) from the sensitive and resistant cells of SW948 and HT29 by FACS sorting using several of the previously reported candidate markers (CD44v6, CD133, EpCAM, and ALDH1) [14,27,35,106,107]. First, we isolated EpCAM+/Ecadherin- cells by FACS analysis. These cells were then sorted for CD44v6+/ALDH1+ cells which were further sorted for CD44v6+/ALDH1+/CD133+ CICs by flow cytometry. Our data show that CD44v6+/CICs overlapped with cells that also expressed the epithelial marker EpCAM, and with CIC markers ALDH1 and CD133 antigen expressions in SW948-FR/SQ cells (Figure 2A–C). In agreement with the results of Figure 1D, which showed that CD44v6 regulated YB-1, we found the colocalization of CD44v6 and YB-1 in the SW948-FR/CICs (Figure 2D). The data in Figure 2E indicate that resistant cells enriched CICs coexpressing CIC markers (EpCAM, CD44v6, ALDH1, and CD133) compared to their sensitive pairs. The coexpression of EpCAM, CD44v6, ALDH1, and CD133 in CICs was validated in four independent samples (Figure 2E). Figure 2F validates the expression of marker proteins in non-CICs and CICs. Collectively, these data in Figure 2 provide evidence that CICs can be prospectively identified and characterized in the CD44v6 (+) CIC cell population, whereas non-CICs are CD44v6 (-) populations, and that CICs coexpress CD44v6 and YB-1, which further validated coregulation of CD44v6 and YB-1 in response to FOLFOX.

### 2.3. Generation of CD44v6 and YB-1 Knockout CICs Using the CRISPR/Cas9 System

To assess the role of CD44v6 and YB-1 in CICs, the CD44v6 and YB-1 genes were knocked out in SW948-FR/CICs using the CRISPR/Cas9 system that has been reported to efficiently disrupt genes in various organisms [108,109]. CD44s (with no alternate splicing) and CD44v6 regions are shown in different colors (Supplemental Figure S2A). Sequence comparison of human CD44s and the CD44v6 isoform are shown in Supplemental Figure S2B. Guide RNAs (gRNA) for CD44v6 genes were designed at exon 6 of CD44 (Supplemental Figure S2C). We obtained two CD44v6 knockout SW948-FR/CICs clones named CD44v6-Mu1 clone (v6 Mu1) and CD44v6-Mu2 clone (v6 Mu2). To design the gRNA for YB-1 we used exon 1 of YB-1 as described previously [110]. Guide RNAs (gRNA) for YB-1 genes are shown in Supplemental Figure S3A. We obtained two YB-1 knockout SW948-FR/CICs clones named YB-1-Mu3 clone and YB-1 Mu4 clone. The data in Supplemental Figures S2D,E and S3B,C demonstrated that the amplified DNA from the gRNA-transfected CICs (v6 Mu1, v6 Mu2, YB-1 Mu3, and YB-1 Mu4) were cleaved into two bands by the T7E1 enzyme (Supplemental Figures S2E and S3C). In contrast, there was no cleaved band for control CICs (Supplemental Figures S2E and S3C). These results indicate that the CD44v6- and YB-1-targeting sgRNA/Cas9 expression plasmids were introduced into the genome of SW948-FR/CICs. After the isolation of single cells from the CD44v6 sgRNA- and the YB-1 sgRNA-transfected SW948-FR/CICs, the CD44v6 gene from the CD44v6-mutated CICs (v6 Mu1 and v6 Mu2) and from the YB-1-mutated CICs (YB-1 Mu3 and YB-1 Mu4) were sequenced. DNA sequencing of v6 Mu1, v6 Mu2, YB-1 Mu3, and YB-1 Mu4 cells revealed deletion of the indicated number of bases in both alleles (Supplemental Figures S2F and S3E). Western blotting (Supplemental Figure S2G) and immunofluorescence (Supplemental Figure S2H) showed that CD44v6 was completely knocked out in v6 Mu1 and v6 Mu2 cells and YB-1 was completely knocked out in YB-1 Mu3 and YB-1 Mu4 cells (Supplemental Figures S3D,F). The data in Supplemental Figures S2 and S3 confirmed that CD44v6 and YB-1 knockout SW948-FR/CICs were generated.

### 2.4. CD44v6 Regulates YB-1 through a PGE2-MTOR Pathway

**Figure 3:**
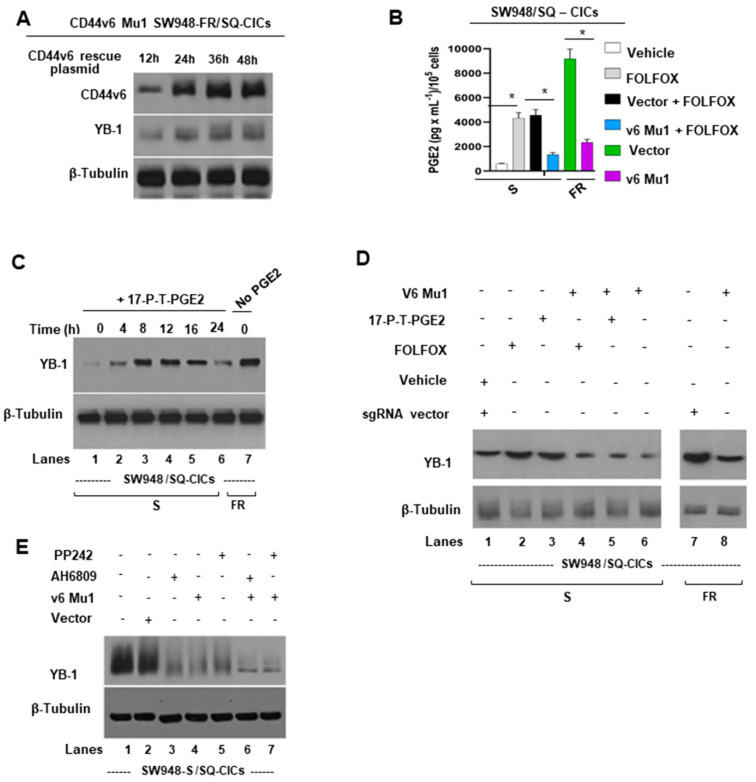
Mechanism of CD44v6 induced YB-1 expression. (**A**) Time course results are shown of CD44v6 and YB-1 protein expressions after transfecting the v6 rescue plasmid (described in the Methods section) into v6 Mu1 SW948-FR CICs. Expression of proteins at 0 h was not shown because of the absence of CD44v6 in the protein lysate. (**B**) Effects of knocking out CD44v6 in in v6 Mu1 CICs of sensitive and resistant SW948 cells on PGE2 production (analyzed by ELISA as described in Methods) in the presence and absence of FOLFOX treatment are shown. (**C**) 17-P-T-PGE2 (synthetic PGE2) induces YB-1 in CICs that were treated with PGE2 at 5 μM for the indicated times. YB-1 expression levels were determined by immunoblotting with anti-YB-1 antibody; β-tubulin as loading control. (**D**) Effects are shown of CD44v6 Mu1 knockout on PGE2 and FOLFOX induced YB-1 expression. CICs isolated from SW948-S and SW948-FR that were previously transfected with either v6 Mu1 or vector for 48 h and then treated with or without synthetic PGE2 or FOLFOX. YB-1 expression levels were determined by immunoblotting with anti-YB-1 antibody; β-tubulin as loading control. (**E**) Effects are shown of PGE2/EP1 receptor, and of mTOR signaling on YB-1 expression. CICs were either transfected with in v6 Mu1 or vector for 48 h and then treated with or without EP1 inhibitor (5 µM AH6809) or mTOR inhibitor (10 nM PP242) for 2 h. They were then cultured in serum-free medium for 16 h and treated with synthetic PGE2 at 5 μM, or 1 × FOLFOX for 24 h. The cell lysates were processed for YB-1 and β-tubulin (as loading control). PGE2 secretion data in Figure 3B represent means ± SD; *n* = 4–6; * *p* < 0.05 compared to either vehicle control, vector + FOLFOX treatment group, or vector control group.

Given that shRNA suppression of CD44v6 protein significantly blocked the FOLFOX-induced YB-1 (Figure 1D), and that the involvement of CD44/CD44v6 in regulating COX2 derived PGE2 production [50,102], which in turn regulates YB-1 in other cancer cell types [111], we investigated a possible interconnection between FOLFOX induced CD44v6-PGE2 signaling and YB-1. First, the v6 Mu1 CICs were transfected with a vector expressing a CD44v6 rescue plasmid in order to rescue CD44v6, and then the expression of CD44v6 and its regulation on YB-1 expression were assessed. Protein analysis by Western blot (WB) showed that CD44v6 protein expression was rescued in v6 Mu1 plus v6 WT CICs (Figure 3A). Importantly, the rescue of CD44v6 also rescued substantial levels of expression within 24–36 h (Figure 3A).

Second, to address the mechanism of CD44v6/PGE2 regulation of YB-1 in CICs, we first evaluated how the CD44v6-PGE2 pathway is affected in FOLFOX resistant SW948-FR CICs. To address this, the levels of secreted PGE2 in the culture media of v6 Mu1, or v6 wild-type (WT) CICs with or without FOLFOX therapy were examined using an ELISA assay. The results (Figure 3B) show that FOLFOX treatment induced significant secretion of PGE2 in SW948-S CICs, and this induction of PGE2 was significantly decreased in v6 MU1 CICs of SW948-S cells. Similarly, v6 MU1 CICs showed substantial reduction in PGE2 production compared to v6 WT CICs of SW948-FR cells. These data (Figure 3B) indicate that a CD44v6/PGE2 signaling axis may have an important role in FOLFOX resistance.

Third, to further explore the mechanism of CD44v6 regulation of YB-1 in CICs, SW948-S CICs were treated with synthetic PGE2 (17-P-T-PGE2) for the indicated times shown in Figure 3C, and PGE2 greatly increased the expression level of YB-1 with time. This stimulation was apparent after 4 h, peaked at 12–16 h (Figure 3C, lanes 4–5 vs. lane 1) and decreased to basal level at 24 h (Figure 3C, lane 6 vs. lanes 4–5) probably due to the depletion of PGE2 in the medium. Importantly, SW948-FR CICs demonstrated constitutively high expression of YB-1 without exogenous addition of PGE2 (Figure 3C, lane 7 vs. lane 1). Next, we found that knockout of CD44v6 protein (v6 Mu1 significantly blocked both the FOLFOX-induced (Figure 3D, lane 4 vs. lane 2) and the PGE2-induced YB-1 expressions (Figure 3D, lane 5 vs. lane 3). These findings indicate that CD44v6 was able to upregulate YB-1 expression through PGE2 in CRC CICs after FOLFOX therapy (Figure 3D, lanes 4 or 5 vs. lanes 2 or 3), or in FOLFOX resistant SW948-FR CICs (Figure 3D, lane 8 vs. 7). Since PGE2/EP1/mTOR promoted YB-1 expression in other cancer cell types [111], we investigated whether PGE2/EP1 receptor and mTOR are also involved in the CD44v6 induced YB-1 expression in SW948-FR CICs. Figure 3E shows that SW948-FR CICs strongly express YB-1 (lanes 1, 2) that is greatly inhibited in v6 Mu1 cells (lane 4 vs. lane 2) and by treatment with EP1 inhibitor AH6809 (lane 3 vs. lane 1), or with mTOR inhibitor PP242 (lane 5 vs. lane1). Moreover, v6 Mu1 SW948-FR CICs treated with EP1 inhibitor AH6809 (Figure 3E, lanes 6 vs. lane 3) or mTOR inhibitor PP242 (Figure 3E, lane 7 vs. lane 5) further inhibited the YB-1 expression more than single treatment (lanes 6, 7 vs. lanes 3, 5). These observations indicate CD44v6 regulated mTOR has important roles in YB-1 expression induced by PGE2/EP1 in response to FOLFOX resistance.

### 2.5. CD44v6-YB-1 Signaling Defines the Stemness of CICs

**Figure 4:**
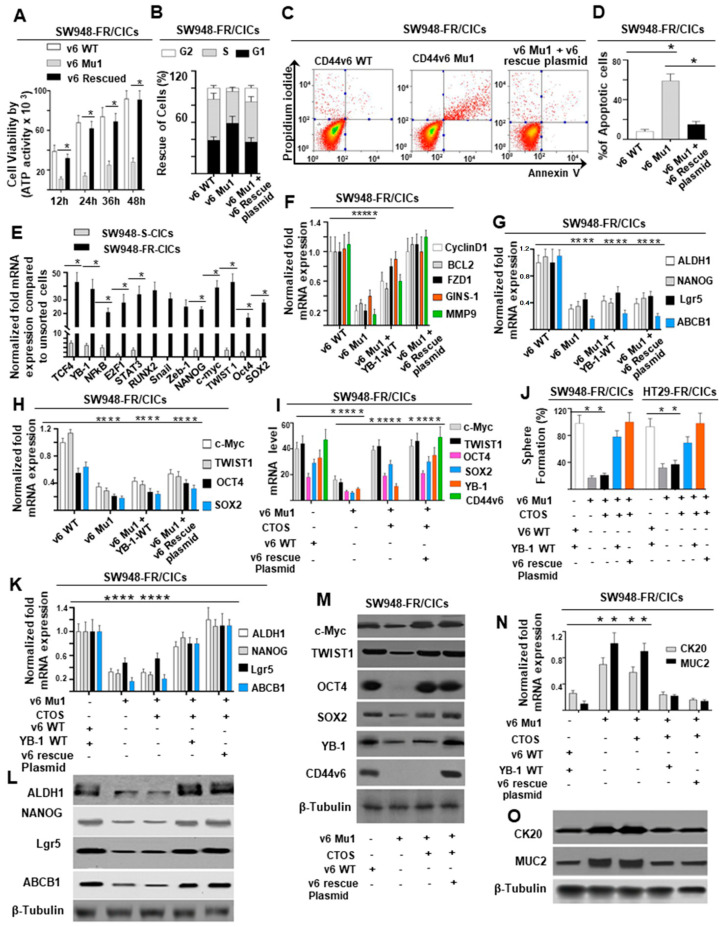
Requirement of CD44v6 and YB-1 for the stemness of SW948-FR CICs after acquisition of FOLFOX resistance. (**A**) The effects are shown of CD44v6 knockout on cell viability (using ATP Glo method) of CD44v6 Mu1, CD44v6 WT, and on CD44v6-rescue SW948-FR/CICs (1 × 10^3^ cells/well of 96-well plate) which were cultured for the indicated times. The time point 0 h represents the number of inoculated cells (data not shown). (**B**) The influence of CD44v6 on cell cycle of CD44v6 Mu1, CD44v6 WT, and CD44v6-rescue SW948-FR CICs are shown. Cells (1 × 10^4^) were cultured for 48 h, and the percentage of cells in G1, S, and G2 phases of cell cycles were examined with flow cytometry. (**C**,**D**) Indicated SW948-FR CICs were seeded into a 6-well plate at 1 × 10^5^ cells/well and cultured for 48 h. The extents of apoptosis of CICs in the cultures were examined by flow cytometry. (**E**) A ChIP assay was performed with chromatin from SW948-FR CICs using an anti-CD44v6 antibody. The immunoprecipitated DNA was amplified by PCR and subcloned. A total of 13 clones were sequenced. Computer-based analysis revealed the presence of various consensus binding sites for common transcription factors in these DNA sequences (see Supplemental Table S2). QPCR analyses show the expressions of these 13 transcription factors in CICs of “S’ and “FR” cells of SW948. (**F**) Expressions are shown for antiapoptosis/stemness-related genes in v6 WT CICs, v6 Mu1 CICs, YB-1 WT overexpressed v6 Mu1 CICs, and in CD44v6 rescue plasmids overexpressed v6 Mu1 CICs. QPCR was conducted to detect the expression levels of the genes. (**G**,**H**) Influence of v6-rescue plasmid into v6 Mu1 CICs for stemness-related gene expressions (**G**) and stemness-related TFs (c-Myc, TWIST1, OCT4, and SOX2) (**H**) were measured by QPCR. (**I**,**M**) The CD44v6-WT plasmid, or the constructs of the “CTOS” (c-Myc, TWIST1, OCT4, and SOX2) TFs either alone or with CD44v6-rescue plasmid, were transfected into the v6 Mu1 SW948-FR CICs. At 72 h after transfection, QPCR (**I**) or Western blot (**M**) analyses were done to examine the expression levels of these transcription factors. (**J**,**K**,**L**,**N**,**O**) Effects of simultaneous expressions of the “CTOS” with the CD44v6-rescue plasmid are shown on sphere formation (**J**), on the expressions of indicated stemness-related genes and protein (**K**,**L**), and on differentiation-related genes and protein (**N**,**O**) in v6 Mu1 SW948-FR CICs. The experiments were biologically repeated for three times. QPCR data represent means ± SD; *n* = 4–6; * *p* < 0.05 compared to vector control, WT control, or vehicle cell control group. Western blot data are representative of three experiments.

Therapeutic resistance has been reported to be a defining feature of CICs [27,112,113,114]. A few recent studies have shown that CD44v6, or YB-1 individually stimulate the drug-resistance in CRC [27,95]. However, the role of CD44v6-YB-1 signaling in stimulating FOLFOX resistance in CRC is unknown. To explore the role of CD44v6 in CICs, the proliferation and viability of v6 WT CICs, v6 Mu1 CICs, and v6 Mu1 CICs plus CD44v6 rescue plasmid, were evaluated. The results of ATP Glo assays revealed that the viability of v6 Mu1 SW948-FR CICs was significantly decreased compared with that of CD44v6 WT SW948-FR CICs (Figure 4A). Rescue of CD44v6 expression in v6 Mu1 CICs led to viability similar to that of v6 WT CICs (Figure 4A). These data indicate that CD44v6 promoted the proliferation of CRC CICs. Next, the cell cycle of CICs was characterized by flow cytometry, and the results in Figure 4B show that the percentage of v6 MU1 CICs in G1 phase was significantly increased compared with that of the corresponding WT CICs. Importantly, when the expression of CD44v6 was rescued in v6 MU1 SW948-FR CICs, the percentage of cells in G1 phase was similar to that of wild-type CICs (Figure 4B).

To characterize the influence of v6 Mu1 knockout on the apoptosis of CICs, apoptotic activities of v6 Mu1, v6 WT, or v6 rescue in v6 Mu1 CICs were examined using Annexin V assays by flow cytometry. The results showed that v6 Mu1 led to a significant increase in apoptotic activity in SW948-FR CICs compared with that in v6 WT cells, while apoptotic activity in v6 rescued Mu1 CICs was closely resembling that in v6 WT cells (Figure 4C,D). The Annexin V data indicated that v6 Mu1 knockout in CICs promoted the apoptosis of CICs (Figure 4C,D).

To explore the mechanism underlying the requirement of CD44v6 for the stemness of CICs, the DNA sequences bound by nuclear CD44v6 complexes were analyzed by ChIP assays in CICs from sensitive and resistant cells of SW948 using a CD44v6-specific antibody. DNA fragments bound by nuclear CD44v6 complexes were pulled down by the anti-CD44v6 antibody from a total of 15 clones. A National Center for Biotechnology Information basic local alignment search tool analysis shows that these clones contained sequences/binding elements for transcription factors (TFs) (Figure 4E; (Supplemental Table S2)) associated with 5-motifs (cell-survival, proliferation, anti-apoptosis, invasion, and stemness) containing genes (CyclinD1, BCL2, MMP9, FZD1, and GINS1) (Figure 4F) that are involved in cell survival, cell proliferation, apoptosis resistance, invasion, and stemness in CICs. Moreover, expressions of these TFs were either very negligible or null in sensitive cell CICs compared to resistant cell CICs (Figure 4E). The promoter sequences associated with TFs (Supplemental Table S2) that bound to the CD44v6 protein could be classified into two themes: (1) genes for 5-motifs such as, Cyclin D1 for cell proliferation/apoptosis-resistance/stemness [115], BCL2 for cell survival [115], FZD1 for stemness [116,117], GINS-1 for DNA replication modulation to regulate proliferation in CICs [118], and MMP9 for cell invasion [119]; (2) four core-stemness genes (ALDH1, NANOG, Lgr5 and ABCB1) for drug-resistance and maintenance of stemness of CRC CICs [115]. The quantitative real-time PCR (QPCR) results showed that the mRNA levels of Cyclin D1, BCL2, FZD1, GINS-1, and MMP9 were significantly increased with rescue of CD44v6 expression in v6 Mu1 SW948-FR CICs (Figure 4F), suggesting that the CD44v6 was responsible for the expression of these 5-motifs related genes. Like other cell surface receptors, CD44v6 is known to migrate to the nucleus as an intact polypeptide or as a proteolytic fragment with or without their ligands. Nuclear localized receptors have been shown to act as co-transcription factors to regulate genes like Cyclin D1 [120,121], FGF2 [122], COX2 [123], c-Jun [124], BCL2 [121], CD49f [96], and MMP9 [125] by interacting with various transcription factors including STAT3 [120,121], RUNX2 [125], NFkB [126,127], p300 [121], E2F1 [128], and YB-1 [96,129]. Although other groups showed binding of nuclear CD44 to chromatin [121,125], our study is the first to demonstrate that posttranslational modification of CD44 is required for efficient interaction between nuclear CD44v6 with the multiple transcription factors (E2F1, YB-1, STAT3, NFkB, and RUNX2; (Supplemental Table S2)) to induce Cyclin D1, BCL2, MMP9, FZD1, and GINS1 promoters. Therefore, in this study, we demonstrate that CD44v6 once engaged with YB-1 is translocated to the nucleus, where it binds to various promoters of genes for 5-motiffs (CyclinD1, BCL2, MMP9, FZD1, and GINS1) in order to maintain CIC growth.

CICs maintain self-renewal to form matching daughter cells by cell division and differentiate into multilineage cells present within tumors [10]. These differentiated cells differ from their normal counterpart by maintaining their malignancy by expressing tissue-specific stemness-marker protein profiles via various core-stemness associated TFs, which define the phenotype of the cells [71]. Recent studies showed that core-stemness associated TFs, including OCT4, KLF4, SOX2, and c-MYC (also known as OKSM), dictate key functions in gastrointestinal tumorigenesis, by regulating cell migration, metastasis, and resistance to therapy [79,130,131]. However, their specific roles in CRC have not been revealed in every aspect so far, particularly with respect to CD44v6 regulation. Of four core transcription factors (CTOS) (c-Myc. TWIST1, OCT4, SOX2), TWIST1 is described to be essential for CRC propagation [85,132], and c-Myc, OCT4, and SOX2 are sufficient to fully reprogram differentiated gastrointestinal cells to gastrointestinal stem cells [133]. In addition, we found that, the DNA:nuclear CD44v6 clones contained cis-regulatory elements c-Myc, TWIST1, OCT4, and SOX2 (Supplemental Table S2), which can define the phenotype of cells of specific tissue, by expressing stemness-related cell surface markers (ALDH1, NANOG, Lgr5, and ABCB1) [84,85,134,135,136] of CICs. Indeed, QPCR data showed that four (ALDH1, NANOG, Lgr5, and ABCB1) stemness-related genes [84,85,134,135,136] were significantly downregulated in v6 Mu1 SW948-FR CICs, respectively (Figure 4G). Rescuing the expression of the CD44v6 protein (v6-rescue) did not restore these inhibitions (Figure 4G). Similarly stemness-related TFs (CTOS) (c-Myc [137], TWIST1 [85], OCT4 and SOX2 [84,138]) were also downregulated in v6 Mu1 SW948-FR CICs, respectively (Figure 4H). These data indicate that these CTOS TFs and the stemness-related genes (ALDH1, NANOG, Lgr5, and ABCB1) as well as CD44v6 were essential for the reversion of differentiated cancer cells.

To restore the differentiated cancer cells into CICs, expression vectors expressing c-Myc, TWIST1, OCT4, and SOX2 (CTOS) were separately transfected into v6 Mu1 SW948-FR CICs with or without a v6-rescue plasmid to express these genes and proteins (shown in Figure 4I,M). Consistently, YB-1 is regulated by CD44v6 (Figure 4I,M). The results in Figure 4J showed that the tumor sphere formation capacities of SW948-FR CICs or HT29-FR CICs were significantly decreased in the absence of CD44v6, even if the CTOS were expressed. Nevertheless, in simultaneous expression of CD44v6 and the CTOS, the tumor-sphere forming capacity of v6 Mu1 knockout CICs was comparable to that of wild-type CICs (Figure 4J). Quantitative real-time PCR analysis showed that the expressions of four stemness genes and proteins (ALDH1, NANOG, Lgr5, and ABCB1) were significantly downregulated in v6 Mu1 SW948-FR CICs even when the CTOS were overexpressed (Figure 4K,L). However, the simultaneous expression of CTOS withYB-1 WT plasmid, or CTOS with CD44v6 rescue plasmid promoted the expression of stemness genes and proteins in v6 Mu1 SW948-FR CICs, which was consistent with the results in v6-WT SW948-FR CICs (Figure 4K,L). These data indicate that CD44v6 and the other CTOS TFs are capable of reverting the differentiated cancer cells into CICs.

To further evaluate the effects of the CTOS on the dedifferentiation of v6 Mu1 knockout CICs, the expression levels of differentiation genes in CRC (cytokeratin 20 (CK20) and mucin 2 (MUC2) [139]) were examined. The results revealed high expression levels of CK20 and MUC2 genes and proteins in v6 Mu1 CICs (Figure 4N,O). Overexpression of the CTOS in v6 Mu1 CICs generated similar results (Figure 4N,O). However, with simultaneous expression of CTOS with YB-1 WT plasmid, or CTOS with CD44v6 rescue plasmid in v6 Mu1 CICs, the expression profiles of differentiation (CK20 and MUC2) genes were consistent with those in CD44v6 wild-type CICs (Figure 4N,O). These results indicate that CD44v6 was required for the reversion of differentiated cancer cells into CICs with stemness.

### 2.6. Analysis of CIC Stemness Associated Genes and Drug-Resistance Proteins in SP Cells

Side population (SP) cells are a subset of enriched progenitor cells with CIC-like phenotypes that exhibit the ability to self-renew as well as give rise to differentiated tissue cells with a distinct low Hoechst 33342 dye staining pattern [140,141,142]. In order to determine whether CD44v6 affects the population of CRC cells, we investigated this aspect by flow cytometry.

**Figure 5:**
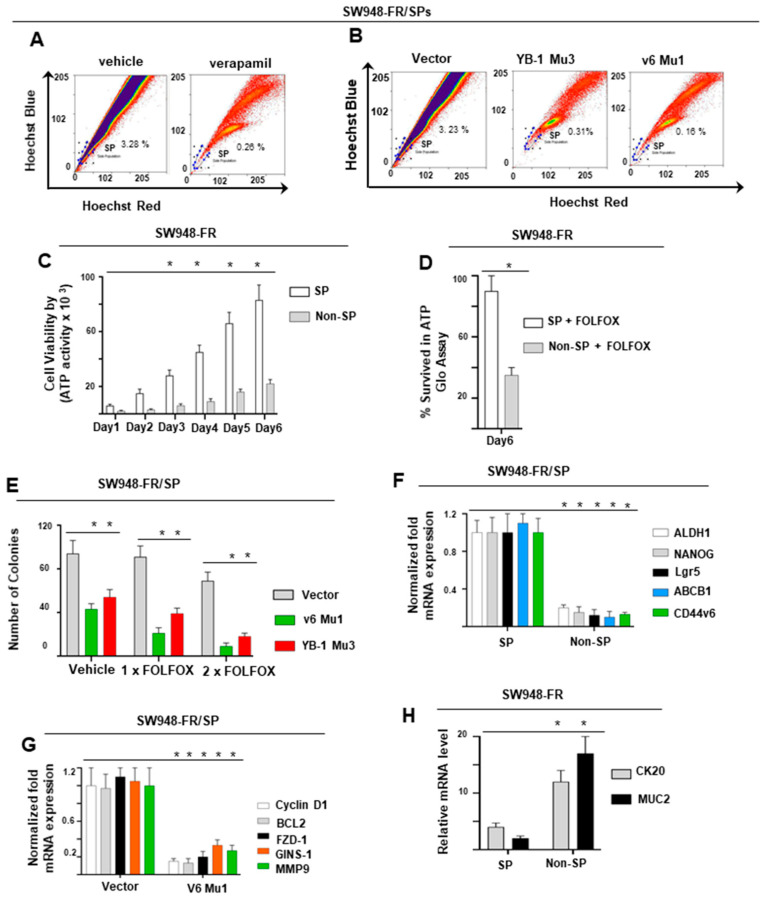
CD44v6-YB-1 defines CIC-like SP cells. (**A**) SW948-FR cells labeled with Hoechst 33342 showed 3.3% of SP cells in the SP gated region. Following treatment with verapamil, the SP cells were reduced to 0.26%. (**B**) v6 Mu1 and YB-1 Mu3 regulate the side population. The SP cells were <10% of vector cells in the v6 Mu1 cells, and the YB-1-Mu3 SW948-FR cells. (**C**) Cell proliferation rates were measured by ATP GLO assay for SP and non-SP cells. SP cells underwent rapid proliferation compared with non-SP cells. (**D**) SP cells exhibited high resistance to 1 × FOLFOX whereas the non-SP cells were sensitive to 1 × FOLFOX. (**E**) CD44v6 and YB-1 knockdown in SW948-FR cells decreased the drug resistance. Control, v6 Mu1, and YB-1 Mu3 SW948-FR cells were treated with various doses of FOLFOX for 10 days in 3% FBS DMEM. Cell viability was assessed using the clonogenic assay. The clonogenicity of CD44v6-Mu1 cells was significantly decreased compared with YB-1-Mu3 cells. (**F**) Expressions of core stemness genes in SW948-FR/SP and non-SP cells by QPCR are shown. (**G**) Expressions of anti-apoptosis/stemness-related genes in vector and v6 Mu1 transfected SW948-FR/SP cells are shown. (**H**) Expressions of CRC differentiation genes in SW948-FR/SP and non-SP cells are shown. Each bar represents the means of three determinations ± SD. * *p* < 0.05 among the indicated groups compared to respective control group. FACs data are representative of three experiments.

We isolated v6-Mu1 SW948-FR, and YB-1 Mu3 SW948-FR single clones, and v6 Mu1 plus YB-1 Mu3 SW948-FR single clones targeted by CD44v6-sgRNA (v6 Mu1), YB-1-sgRNA (YB-1 Mu3) expression plasmids. We cultured individual clones each in 96-well plates and allowed them to grow by changing the media 3× times a week until colonies were formed. After dissociation of single clones, we cultured them in 24-well tissue culture plates, and further cultured them in 6-well plates to get increased cell numbers. These cultures were purified by flow cytometric analysis using a CK (pan)-fluorescein isothiocyanate antibody. Flow cytometric analyses were done with SW948-FR cells after treatment with verapamil, or from v6 Mu1, or YB-1 Mu3 clones. The cells were stained with Hoechst 33342 (Figure 5A,B). The levels of SP cell populations were substantially diminished in the presence of verapamil or from v6 Mu1, and YB-1 Mu3 clones. Our results (Figure 5B) also suggest that a significantly higher population of SP cells were reduced with v6 Mu1 (0.15%) compared to YB-1 Mu3 cells (0.31%). Purified isolated SP cells and non-SP cells from various sgRNA transfectants of SW948-FR cells were cultured separately and grown in fresh medium for 2 weeks. Afterwards, cultured SP cells and non-SP cells were subjected to flow cytometric analysis using Hoechst 33342 dye again to reanalyze these SP and non-SP populations. The freshly sorted SP were further processed for cell viability/cell proliferation analysis using ATP Glo and clonogenic assays. The data shows that SW948-FR-SP cells demonstrated substantially increased cell proliferation compared to non-SP cells (Figure 5C, ≈ 5 fold at day 6). Consequently, the SP cells were analyzed for FOLFOX-sensitivity in chlonogenic growth assay. The results in Figure 5D show that 1 × FOLFOX sensitizes the non-SP cells (34% survival) whereas the SP-cells are resistant to apoptosis/death (Figure 5D, 91% survival), suggesting that the SW948-FR SP cells are highly resistant to FOLFOX.

Results from Figure 5E demonstrate that CD44v6 and YB-1 downregulation sensitizes SW948-FR/SP cells to 1 × FOLFOX and more to 2 × FOLFOX treatment. Knockdown of CD44v6 led to increased cytotoxicity compared to YB-1 Mu3 SW948-FR/SP cells in response to 1 × FOLFOX and to 2 × FOLFOX treatment with reduced colony formation (Figure 5E). The residual colony number after knockdown of CD44v6 and YB-1 (Figure 5E) suggests that in addition to CD44v6 regulation of YB-1, YB-1 may also be regulated by other signaling. Thus, CD44v6-YB-1 signaling activity mediates FOLFOX resistance in resistant SP cells.

A recent study demonstrated that overexpression of the drug resistant transporter contributes to Hoechst dye expulsion and the drug-resistance properties of SP cells in solid tumors including CRC cells expressing stemness genes [143,144]. We analyzed the stemness gene expressions of ALDH1, NANOG, LGR5, ABCB1, as well as CD44v6 in SP and non-SP cells from the SW948-FR cells. QPCR analysis revealed that these stemness genes were more highly expressed in SP cells than in non-SP cells (Figure 5F). Subsequently, mRNA analysis revealed that the protein expression of cell proliferation/apoptosis-resistance/stemness-related genes (CyclinD1, BCL2, FZD1, GINS-1, MMP9, and MDR1) were significantly decreased in v6 Mu1 SP cells (Figure 5G). To further evaluate the role of the dedifferentiation of non-SP cells versus differentiation of SP cells, the expression levels of differentiation genes (CK20, MUC2) in SP and non-SP cells of SW948-FR cells were examined. The results revealed high expression levels of these differentiation genes in non-SP cells compared to SP cells (Figure 5H). These findings clearly demonstrate that elevated expression of CD44v6, YB-1, and MDR1 (ABCB1) as well as other stem cell and antiapoptotic genes/proteins are likely responsible for the FOLFOX apoptotic resistance, self-renewal capacity, and rapid proliferation, and for the reversion of differentiated cancer cells into CICs.

### 2.7. Nuclear YB-1 Associates with CD44v6 and Functions to Modulate CD44v6 and MDR1 Transcription

**Figure 6:**
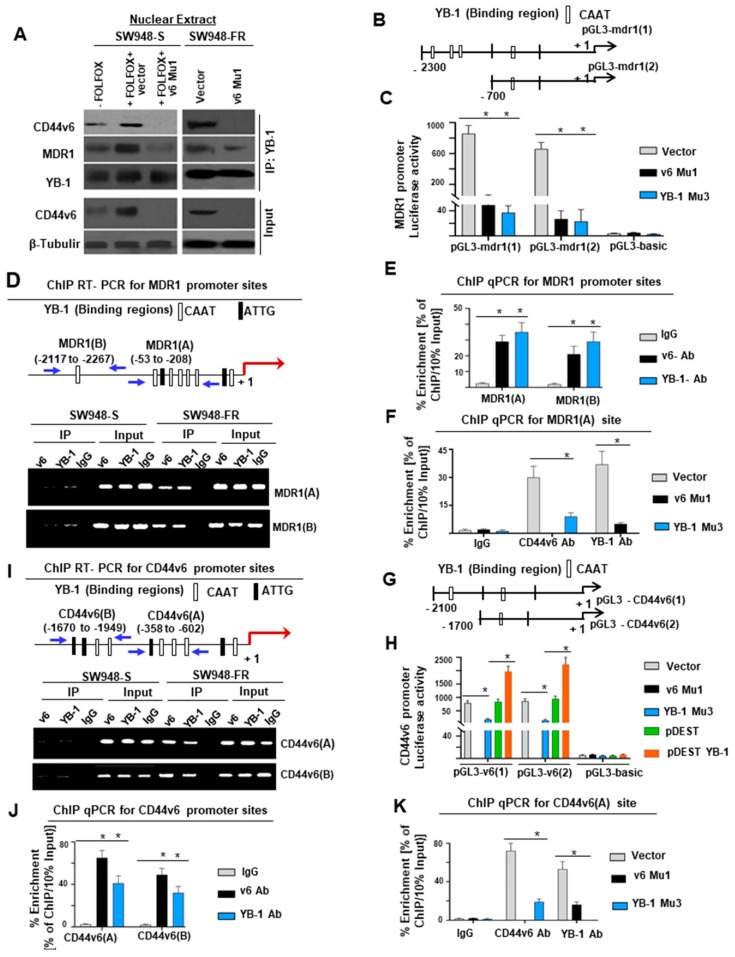
Nuclear YB-1 binds with CD44v6 and modulates CD44v6 and MDR1 transcription by binding to CD44v6 and MDR1 promoters. (**A**) Nuclear extracts were isolated from SW948-S and SW948-FR cells first transfected with vector, or v6 Mu1, and then treated with or without 1 × FOLFOX for 8 h. Nuclear extracts were immunoprecipitated by YB-1 antibody followed by Western blotting of the indicated proteins. (**B**) The scheme shows the MDR1 promoter constructs with YB-1 binding sites (mdr1(1) and mdr1(2)). (**C**) MDR1 Luciferase activities are shown for SW948-FR/CICs overexpressing CD44v6 Mu1, or YB-1 Mu3, or vector (Control) for 24 h. (**D**,**F**) MDR1 is transcriptionally regulated by YB-1 in SW948-FR/CICs. (**D**) The sketch map shows the predicted YB-1 binding sites (CAAT or ATTG) within the MDR1 promoter (MDR1(A) and MDR1(B)). PCR primers designated for MDR1(A) and MDR1(B) were used for amplification of the potential YB-1 binding sites of the MDR1 gene by ChIP semiquantitative PCR assays using anti-CD44v6, anti-YB-1, or an irrelevant IgG antibody (control). Total genomic DNA was used as input for the ChIP PCR. (**E**) ChIP QPCRs representing the PCR products in CD44v6, YB-1, or IgG immunoprecipitated DNA versus 10% input DNA in SW948-FR/CICs using primers for MDR1(A) and MDR1(B) sites are shown. (**F**) ChIP QPCRs representing the PCR products in CD44v6, YB-1, or IgG in SW948-FR/CICs overexpressing CD44v6 Mu1, or YB-1 Mu3, or vector for 24 h are shown. (**G**) The sketch map of predicted YB-1 binding sites (CD44v6 [1] and CD44v6 [2]) within the CD44v6 promoter is shown. (**H**) CD44v6 luciferase assays are shown for SW948-FR/CICs overexpressing v6 Mu1, or YB-1 Mu3, or vector for 24 h. (**I**) Semiquantitative PCR products using ChIP QPCR primers are shown for designated YB-1 binding sites as CD44v6(A) and CD44v6(B). (**J**) A representative ChIP QPCR representing the PCR product in immunoprecipitated CD44v6 is shown. (**K**) ChIP QPCR using PCR primers for designated CD44v6(A) sites were used for amplification of the YB-1 binding sites of the CD44v6 gene in ChIP assays in untreated CICs, or CICs overexpressing CD44v6 Mu1, or YB-1 Mu3, or vector (Control) for 24 h. Values represent means ± SD; *n* = 3–5; * *p* < 0.05, compared to whole cell lysate and nuclear fractions isolated from sensitive cells, sensitive cell groups, vehicle control, IgG control, vector control, and appropriate control groups. Western blot and semiquantitative PCR data are representative of three experiments.

A recent study demonstrated that YB-1 regulated CD44 in primary breast cancer cells [96]. Moreover, YB-1 also regulates MDR1 in primary breast cancer cells [99]. To understand the mechanism of YB-1-CD44v6 regulation, chromatin immunoprecipitation (ChIP) was done to identify DNA sequences bound by nuclear CD44v6 complexes. DNA fragments were pulled down by anti-CD44v6 antibody from a total of 15 clones. A National Center for Biotechnology Information basic local alignment search tool analysis shows that these clones contained sequences (Supplemental Table S2) corresponding to the promoters of several genes, including β-catenin. Among them, 13 clones contained sequences for YB-1. Thus, we tested whether nuclear CD44v6 exerts its transcriptional regulatory function on MDR1 through interacting with YB-1. Coimmunoprecipitation (Co-IP) showed nuclear colocalization of CD44v6 and YB-1 in unstimulated SW948-FR and FOLFOX stimulated SW948-S nuclear extracts (Figure 6A). Our data also showed that very little CD44v6 and YB-1 were associated with YB-1 immunoprecipitates (IPs) of nuclear extracts of SW948-S cells, whereas elevated expressions of CD44v6 and YB-1 were found with YB-1 IP in the nuclear extracts of SW948-FR cells that endogenously express high levels of CD44v6 and YB-1 (Figure 6A). Knocking down CD44v6 in v6 Mu1 CICs significantly suppressed expression of CD44v6 and YB-1 in nuclear lysates of SW948-FR cells (Figure 6A).

To further evaluate the relative contribution of the YB-1 transcription factor to the regulation of MDR1 promoter activity, we performed transient transfection assays using SW948-FR cells with constructs containing YB-1 binding sites within the MDR1 promoter (Figure 6B) cloned into a luciferase reporter plasmid. These PGL3-mdr (1) and PGL3-mdr (2) constructs were transfected with or without manipulations of CD44v6 and YB-1, and luciferase activities were measured. The results showed that luciferase activity increases in the presence of YB-1 binding sites in these SW948-FR CICs (Figure 6C). Even with only one YB-1 binding site in the pGL3-mdr1 (2), the activity was only slightly less than pGL3-mdr (1) with more than one YB-1 binding site (Figure 6C). The MDR1 promoter luciferase constructs negatively responded to cotransfection with v6 Mu1 and YB-1 Mu3 in SW948-FR CICs (Figure 6C). These reductions show that YB-1 promoter binding and activation of MDR1 is mediated through both CD44v6 and YB-1 in the nucleus of SW948-FR CICs.

To identify whether CD44v6 binds to YB-1 binding sites in the MDR1 promoter in SW948-FR CICs, ChIP assays were done. Immunoprecipitated YB-1-, CD44v6-, and IgG-chromatin complex and input DNA were amplified using primers (Supplemental Table S3) covering the indicated YB-1 binding sites of the MDR1 promoter (as shown in Figure 6D). Semiquantitative ChIP RT-PCR assays (Figure 6D) showed that YB-1 bound to two MDR1 sites, and expression levels were almost null in sensitive SW948-S cells and significantly increased in resistant SW948-FR cells. CD44v6 only bound to these two sites in resistant cells with markedly increased binding to YB-1 when compared to very less or no association with sensitive cells (Figure 6E). Knockdown of CD44v6 and YB-1 reduced endogenous *MDR1* promoter binding to CD44v6 and YB-1 in DNA complexes in SW948-FR CICs (Figure 6F), thus validating our results from luciferase reporter assays that CD44v6 and YB-1 coregulate MDR1 expression in a CD44v6-regulated manner in FOLFOX resistant SW948-FR CICs.

Several putative YB-1 binding sites were located 2.1 kilobases upstream of the transcriptional start site of the *CD44v6* gene (Figure 6G). A fragment of the *CD44v6* gene promoter (−2100 to 500 bp) was fused upstream of the firefly luciferase gene in pGL3-CD44v6 (1), and similarly in pGL3-CD44v6 (2) it was fused upstream (−1700 to 500 bp). pGL3-CD44v6 (1) and pGL3-CD44v6 (2) contain YB-1 binding sites. Luciferase assays were used to directly examine the interaction between YB-1 and the *CD44v6* promoter. The luciferase activities in SW948-FR cells transfected with v6-Mu1 and YB-1 Mu3 SW948-CICs were significantly lower than vector control group, while YB-1 overexpression significantly increased the luciferase activity (Figure 6H). This provides evidence that YB-1 increases CD44v6 transcription activity. To validate these results, conventional ChIP analyses were done, and they provided direct evidence for the ability of YB-1 to bind to the promoters of CD44v6 (Figure 6I–K). Thus, the results in Figure 6 show that a YB-1 pathway promotes both CD44v6 and MDR1 gene expressions. Overall, the above results indicate that FOLFOX treatment mediates a CD44v6-PGE2-mTOR-YB-1 pathway that promotes CD44v6 expression (Figure 3 and Figure 6) and functions through a positive feedback loop between CD44v6 and YB-1 that activates MDR1 gene expression and CD44v6 splicing through noncanonical signaling and thereby mediates FOLFOX resistance.

### 2.8. Role of CD44v6-YB-1 in the Tumorigenesis of CICs of Resistant Cells In Vivo

**Figure 7:**
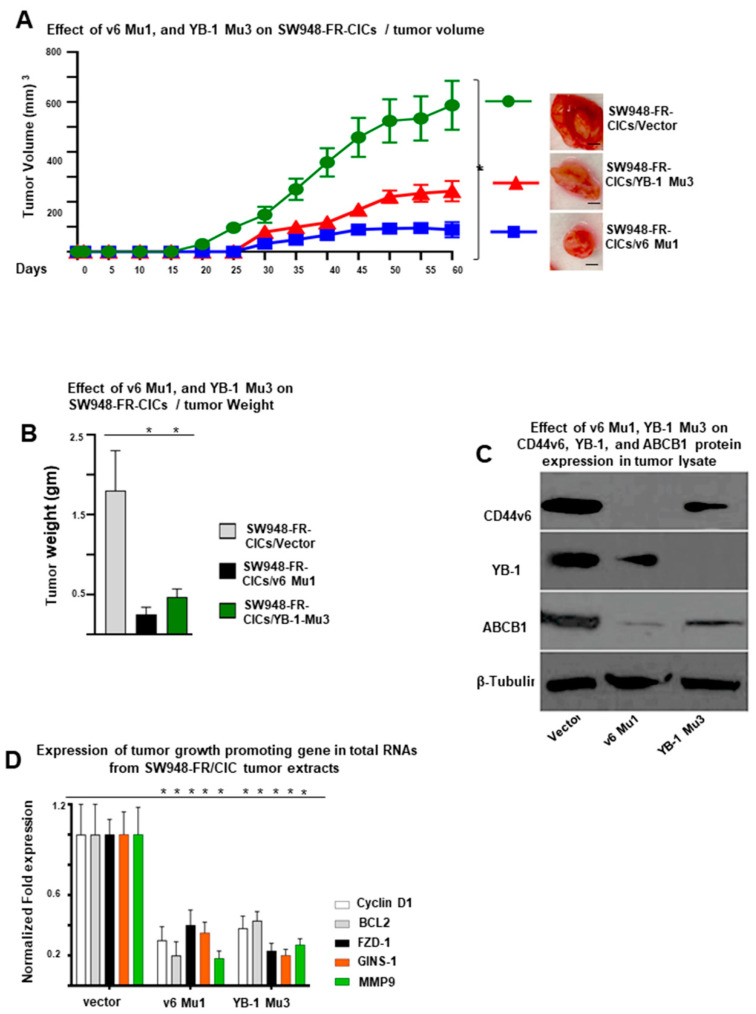
Role of CD44v6 in the SQ tumorigenesis of SW948-FR/CICs in vivo. (**A**,**B**) Effects of CD44v6, or YB-1, or a combination of CD44v6 + YB-1 knockout on tumor growth in nude mice are shown. CD44v6 Mu1, YB-1 Mu3, or v6 Mu1 + YB-1 Mu3 knockout FOLFOX resistant CRC CICs and wide-type CICs were injected into nude mice. The tumor volumes in mice were measured every five days (**A**). Sixty days later, the mice were sacrificed. A solid tumor was collected from each mouse. (**B**) The impacts of v6 Mu1, YB-1 Mu3 knockout FOLFOX resistant CRC CICs on tumor weights are shown. (**C**) The CD44v6, MDR1, and YB-1 protein levels in tumors of mice injected with v6 Mu1, YB-1 Mu3 knockout FOLFOX resistant CRC CICs or WT CICs are shown. β-Tubulin was used as an internal control. (**D**) Expressions of proliferation/antiapoptosis/invasion/stemness related genes (by QPCR) in these solid tumors are shown. Data are presented as mean ± SD (*n* = 7); * *p* < 0.05. ANOVA followed by Bonferroni’s post-hoc test was used to assess the significance. Western blot data are representative of three experiments. QPCR data are presented as mean ± SD (*n* = 4); * *p* < 0.05. Scale bar, 50 μm.

To evaluate the impact of CD44v6-YB-1 on the tumorigenesis of CICs in vivo, v6 Mu1 and YB-1 Mu3 knockouts in SW948-FR-CICs and WT CICs, were injected into nude mice. Our results indicated that tumor growth in mice injected with v6 Mu1 CICs and YB-1 Mu3 CICs transplanted tumors were significantly suppressed compared with those in control mice (Figure 7A). The tumors in mice injected with v6 Mu1 CICs were smaller and weighed less than YB-1 Mu3 CICs (Figure 7B). In addition, Western blot data revealed that the CD44v6 and YB-1 proteins were detected lesser in tumors in mice injected with v6 Mu1 CICs or with YB-1 Mu3 CICs (Figure 7C). The residual expression of YB-1, and ABCB1 (MDR1) protein in v6 Mu1 CIC injected tumors (Figure 7C) suggest that in addition to CD44v6-YB-1, other signaling cascades may also mediate the potential of FOLFOX to stimulate YB-1 expression/activity.

These results showed that CD44v6 knockout v6 Mu1 SW948-FR cells inhibited the tumorigenesis of CICs in vivo. As CD44v6 defined CICs autonomous resistance (Figure 4A–D) and has been proven to participate in the transcriptional regulation and activation of stemness factors in resistant SW948-FR CICs and SP cells (Figure 4E and Figure 5D,F), we investigated the effects of CD44v6 knockout on the expression of CD44v6/YB-1 target genes in vivo. The QPCR analysis of the xenograft tumor RNAs showed that the stemness-associated genes were considerably decreased in YB-1 Mu3 knockout tumors compared with those in WT control tumors (Figure 7D). These findings further suggest that CD44v6 promoted the expression of stemness-associated genes including MDR1 to enhance the tumorigenesis of CICs in vivo and that this function of CD44v6 requires a positive feedback loop coupling CD44 alternative splicing and it’s downstream target YB-1 activation.

## 3. Discussion

Classically, CSCs/CICs are most often defined as being multipotent, long-lived, slow cycling/quiescent, self-renewable, and asymmetrically dividing cells within a tumor that have tumorigenic potential when transplanted into immune-deficient mice [10,145,146,147]. Thus, CICs are primarily involved in maintenance of tissue homeostasis and recovery from injury. CICs can be separated from other cancer cells based on their distinctive cell surface markers depending on the cancer of origin [148]. Due to CIC’s self-renewal and multilineage differentiation characteristics, tumors are composed of a biological hierarchy of cell types that is dictated by CICs [149]. As a result of its self-renewing capabilities, CICs can be serially transplanted through multiple generations to relapse the tumor with strong drug-resistance and metastatic traits [38]. While surgical removal and adjuvant therapy can cure well-confined primary tumors, resistant tumors that are metastatic are largely incurable because of the resistance of disseminated tumor cells to existing therapeutic agents of standard care [150,151,152]. Thus, the identification of the molecular mechanisms involved in FOLFOX resistance provides strong impetus to investigate therapeutically tenable cellular/molecular pathways for reversal of resistance.

With regard to cancer mimicking development and wound response outcomes, CICs function in two settings. First, stem cells were initially described in acute myeloid leukemia [153], but soon were also found in solid tumors including colorectal cancers [154]. Single hematopoietic stem cells repopulate to the bone marrow niche via systemic blood circulation, and the niche then directs instructive signals to sustain proliferative potential of stem cells. Second, organ regeneration by single hematopoietic stem cells is less evident in solid tumor tissue. Recent work in CRC has shown that the inner surface epithelium of the colon is folded into crypts where stem cells display a cooperative relationship with supportive CD24+ (or cKit+) accessory cells similar to small intestinal Paneth cells. Paneth cells (CIC niche cells) potentially proliferate significantly relative to single stem cells, and they provide maintenance signals and restrain uncontrolled growth. When a stem cell exits its niche, it undergoes lineage specification and differentiation, providing an essential role for the stromal-environment to influence normal cellular hierarchies [147].

Colorectal CICs with epithelial characteristics, i.e., EpCAM (+) CD44v6 (+) [27,154], share the major intestinal stem cell features, including self-renewal, telomerase activity, organ-specific differentiation potential, unusual activation of proliferating signaling including CD44v6 and WNT/β-catenin, elevated tumorigenic potential, and drug-resistance [155,156,157]. In CRC, tumor cells are closely associated with cancer associated fibroblasts, which secrete different stromal factors, including hepatocyte growth factor, WNT, TGFβ, periostin, prostaglandin E2 (PGE2), bone morphogenetic protein (BMP), and interleukins produced by the tumor microenvironment, which in turn promotes tumor stemness and CIC clonogenicity [13,72,158,159,160]. These factors in turn induce the expression of different transcription factors (TFs), including TWIST1 and Snail. These TFs repress E-cadherin expression, and trigger EMT, which enables cancer cells to disseminate and acquire the ability to self-renew and resist apoptosis [72,134]. For self-renewal and drug resistance function CICs express a distinct set of markers (ABC transporters, CD133, EpCAM, Lgr-5, or ALDH1) [84] as well as their associated TFs, and stemness genes including BIM-1, NANOG [134,135], TWIST, OCT-4 [161,162,163], SOX2 [161,164], and FZD1 [117]. In addition to being CIC markers, these molecules are biologically functional and can be expressed in tumor cells by continuous activation of transcriptional networks that constitutively express high levels of stemness-associated TFs [79,134,135,161,164,165].

In this study, we demonstrated that CRC-CICs express CD44v6, which is a functional marker involved in autonomous resistance of CICs to FOLFOX therapy and was validated by the major inverse correlation between tumor survival and CD44v6 expression. Additionally, we showed that the YB-1 oncogene is widely expressed in CD44v6+ CICs, thus enabling these CICs to be highly resistant to FOLFOX therapy. Further we show for the first time that CD44v6 induces YB-1 through CD44v6 regulated PGE2-mTOR signaling in response to FOLFOX, and that CD44v6-YB-1 promotes tumor sphere, soft agar colony formation, and drug-resistance in CRC-CICs. In addition, we found that FOLFOX resistant cells contained more SP cells compared to the FOLFOX sensitive cells. Furthermore, SP cells highly expressed stemness-related genes and resistance to FOLFOX. Importantly, we found that SP cells in CRC had significantly increased activation of CD44v6 and YB-1 expressions compared to non-SP cells. Knocking down CD44v6 in SP cells significantly decreased stemness related genes and increased differentiation markers. Further, SP cells in FOLFOX resistant CRC cells were highly tumorigenic in vivo compared to non-SP cells. Therefore, these findings indicate that SP cells are likely to be a major driving force of CRC resistance to FOLFOX, indicating that the CD44v6-YB-1 signaling pathway may be an important target for eliminating CICs in CRC.

The CIC subpopulation can differentiate into non-CIC tumor cells and promote phenotypic and functional heterogeneity within the tumor. In 2006, Yamanaka lab showed that terminally differentiated fibroblasts can be reprogrammed to induced pluripotent stem cells (iPSC) using four transcription factors OCT4, SOX2, KLF4, and c-Myc by initiating several synergistic processes [130,166]. Thus, any somatic cell can be reprogrammed into iPSC cells by coexpression of stemness-related core transcription factors that are specific for tumor cell type, and these TFs can act in partnership to regulate the expression of discrete genes specific for maintaining stem cell pluripotency and self-renewal [167,168,169,170]. Such factors specific to each tumor type maintain the dynamic balance between CICs and differentiated cells in a proper equilibrium. Dedifferentiation can alter this equilibrium resulting in metastasis/aggressiveness since CICs are resistant to chemotherapy and radiation. In this study, our results showed that CD44v6 knockout induced differentiated CICs, leading to the decrease of sphere-forming ability and downregulation of stemness genes related to CICs. However, CD44v6 rescue alone in the v6 Mu1 SW948-CICs did not reprogram the differentiated cells into CICs, which was in agreement with the previous studies [79,130]. Our results revealed that v6 Mu1 knockout CICs significantly promoted the expression of differentiation genes (CK20 and MUC2). We also revealed that the simultaneous expressions of c-Myc, TWIST1, Oct 4, and SOX2 (CTOS)), and a v6 rescue plasmid reverted v6 Mu1 CICs into CICs. Therefore, our study showed that differentiated cells can be reprogrammed into CICs via combined expression of CTOS TFs and CD44v6. This indicates that CD44v6 induced YB-1 was required for the reversion of differentiated cancer cells into CICs. Overall, we can conclude that after FOLFOX stimulation, internalized CD44v6 complexes with YB-1, and the combined signaling complex reaches the nucleus, where CD44v6 regulated YB-1 stimulates promoters for the MDR1 and CD44v6 genes, which sustains FOLFOX resistance and tumor formation through CD44v6 overexpressing CICs. This provides evidence that CD44v6 is a biomarker and a likely therapeutic target. In addition, CD44v6-YB-1 interaction could promote CIC proliferation, maintain CIC stemness, and suppress CIC apoptosis by enhancing the expression of TFs (NFkB, E2F1, STAT3, and RUNX2) for proliferation/antiapoptosis/invasion/stemness related promoters (Cyclin D1, FZD1, GINS1, BCL2, and MMP9). Therefore, CD44v6-YB-1 signaling has a vital role in the activation and reversion of the differentiated cancer cells into CICs.

## 4. Materials and Methods

### 4.1. Materials

Dulbecco’s Modified Eagle’s Medium (DMEM), Eagle’s Minimum Essential Medium (EMEM), McCoy’s 5A Medium, F-12K Medium, Leibovitz’s L-15 Medium, L-Glutamine, Sodium pyruvate, Penicillin (100 µ/mL), and Streptomycin (100 µg/mL), sodium pyruvate, 0.05% EDTA solution (Versene), Phosphate buffered saline (PBS, Calcium and Magnesium free), and 0.05% Trypsin were from Corning Inc. (Upstate, NY, USA). Fetal Bovine Serum (FBS) was from Atlanta Biologicals (Minneapolis, MN, USA). Amphotericin B was from Hyclone (Logan, UT, USA). Nonidet P-40, EGTA, sodium orthovanadate, glycerol, phenylmethylsulphonyl fluoride, leupeptin, pepstatin A, aprotinin, and HEPES, Insulin and B-27, Hoechst 33342 were from Sigma (St. Louis, MO, USA). Collagenase was from Worthington Biochemical (Lakewood, NJ, USA). Fc blocking reagent was from Millenia Biotech (Gieβen, Germany). bFGF, Blocking antibody for CD44v6 (2F10), IL17A and isotype control were from R&D Systems (Minneapolis, MN, USA). TrypLE, Lipofectamine 2000 was from Invitrogen (Carlsbad, CA, USA). The 50 μm nylon mesh was from BD Biosciences Thermo Fisher (Waltham, MA, USA). Lipofectamine 2000 was from Invitrogen (Carlsbad, CA, USA). T7E1 was from New England Biolabs (Beverly, MA USA). The antibodies against CD44v6, YB-1, E-Cadherin, EpCAM, ALDH1, NANOG, Lgr5, EpCAM, CD133, β-catenin, Anti-Active-β-catenin (anti-ABC) antibody, clone 8E7, TCF4, MDR1, Cyclin D1, BCL2, FZD1, GINS-1, MMP9, β-tubulin, horseradish peroxidase-linked anti-rabbit and anti-mouse antibodies, and Luminol reagent were purchased from commercial sources (R&D (Minneapolis, MN, USA), Santa Cruz Biotechnology Inc. (Dallas, TX, USA), Abcam (Cambridge, MA, USA), Ebioscience Thermo Fisher, Thermo Fisher (Waltham, MA, USA), and Cell Signaling Technology (Danvers, MA, USA)). Blocking antibody for CD44v6 (2F10), IL17A and isotype control were from R&D Systems. Blocking antibody for periostin (OC-20) was from Adipogen Life sciences (San Diego, CA, USA). Blocking antibody for WNT3A (1H12L14) was from Thermo Fisher (Waltham, MA, USA).

### 4.2. Cell Lines

Human colorectal adenocarcinoma cell lines: (1) HT29 (HTB-38) purchased from ATCC (Manassas, VA, USA)was maintained in McCoy’s 5A medium+ 2mM Glutamine + 10% Fetal Bovine Serum (FBS); (2) pre-neoplastic Apc 10.1 cells isolated from Apc min/+ mice were cultured in Dulbecco’s modified Eagle medium supplemented with 20% FBS and harvested by a 15–30 min treatment with trypsin-EDTA solution [48,171]; (3) Colo 205 and Colo 320DM, SW707, and HCT15 cells purchased from ATCC and Cellosaurus were cultured in ATCC-RPMI 1640 + 2mM Glutamine + 10% FBS; (4) SW620 and SW948 cells purchased from ATCC were cultured in Leibovitz’s L-15 Medium + 2mM Glutamine + 10% FBS. The cell lines were maintained in medium mentioned next to the cell line in humidified atmosphere in the presence of 10% FBS, penicillin (100 µg/mL), and streptomycin (100 µg/mL), 5% CO_2_ at 37 °C.

### 4.3. Generation of FOFOX Resistant (FR) Cells

To determine the mechanisms of FOLFOX resistance, we selected three cell lines (HT29, and SW948) out of seven cell lines (Supplemental Figure S1A), which have low basal levels of CD44v6 gene expression. Using these cell lines, we determined their IC_50_ values for 5-Flourouracil (5-FU) and their IC_50_ values for oxaliplatin (OXA) (Figure S1B–D) because these molecules are the components of FOLFOX. To determine these IC_50_ values, cells were separately pretreated with various concentrations of 5-FU, or OXA, or vehicle. After a 24-h incubation at 37 °C, growth assays were analyzed as described above. The 50% inhibitory concentration (IC_50_) was identified as a concentration of drug required to achieve a 50% growth inhibition relative to untreated controls. The average IC_50_ values for HT29, and SW948 cells for 5-FU is 50 µM and for OXA is 10 µM. FOLFOX resistance cells were generated by incubating the sensitive parental (HT29-S, and SW948-S) cells with increasing concentrations from 1 × FOLFOX (50 µM 5-FU + 10 µM OXA + 1 µM leucovorin) to 5 × FOLFOX over 3 days. The surviving FOLFOX resistant (FR) cells were cultured in normal medium for 5 days. This exposure and withdrawal cycle were repeated five times for each dose of FOLFOX. The resistances of these FR clones were compared to sensitive pairs by determining the number of colonies in soft agar growth with 1 × FOLFOX–5 × FOLFOX therapy.

### 4.4. Isolation of CICs

Our FOLFOX resistant (FR) clones and the corresponding sensitive pairs were maintained through subcutaneous xenografts in the flanks of immunocompromised SCID mice. Fresh normal colonic tissue and colorectal tumors were rinsed in DMEM (Life Technologies) supplemented with 200 units/mL of penicillin, 200 µg/mL of streptomycin, and 4 units/mL of amphotericin B and minced, followed by incubation with 300 units/mL of collagenase at 37 °C for 3 h. A single cell suspension was obtained by filtration through a 40 µm filter. After discarding lymphocytes by gradient centrifugation, the cells were washed twice in fluorescence-activated cell sorting (FACS) buffer (Phosphate-buffered saline (PBS) + 2% BSA + 1 mM EDTA + 0.1% sodium azide), incubated with Fc blocking reagent and stained with directly with conjugated antibodies by incubating on ice for 20 min. They were then sorted in a Mo Flo cell sorter for EpCAM+CD44v6+ALDH1+CD133+ (CD44v6 high CICs) by using appropriate fluorescent conjugated antibodies. They were further confirmed by tumor sphere formation abilities and in vivo tumorigenicities by testing for tumor sphere formation at 37 °C in an atmosphere of 5% CO_2_. CICs were cultured in serum-free medium with basic fibroblast growth factor (bFGF, 10 ng/mL) and epidermal growth factor (EGF, 10 ng/mL), 5 μg/mL of insulin, and 2% of B-27 at 37 °C in a humidified atmosphere with 5% CO_2_. For cell counting, before each experiment, a single-cell suspension was achieved using TrypLE (Invitrogen) dissociation.

### 4.5. Labeling of Cells with Hoechst 33342

Approximately 10^6^ cells/mL from WT, v6 Mu1, YB-1, Mu3 SW948-FR cells in 10% DMEM were labeled with Hoechst 33342 stock bis-benzamide (5 μL/mL) either with dye alone or in combination with drug treatment (verapamil, 0.8 μL/mL). After 90 min incubation in a water bath at 37 °C, cells were subjected to centrifugation at 2500× *g* for 10 min at 4 °C and resuspended in 500 μL of Hank’s balanced salt solution containing 10 mM HEPES (4-[2-hydroxyethyl]-1-piperazineethanesulfonic acid). Finally, cells were counterstained with propidium iodide (2 μg/mL sample) at 4 °C to exclude dead cells. Cells were filtered through a 50 μm nylon mesh to remove cell clumps into labeled fluorescence-activated cell sorting (FACS) tubes. The SP cells and main population (non-SP) cells were sorted using a flow cytometer. The Hoechst 33342 dye was excited at 355 nm, and its dual-wavelength fluorescence was analyzed (blue, 450 nm; red, 675 nm).

### 4.6. Establishment of CD44v6 Knockout Mutant and YB-1 Knockout Mutant of CICs by the CRISPR/Cas9 System

A guide RNA for CD44v6 and a guide RNA for YB-1 were designed using the ZiFit Web application (http://zifit.partners.org/) to aim at exon 6 of *CD44*, and at exon 1 of *YB-1*. The gene-specific guide RNA sequence for CD44v6 was 5′-GGGGTAGGGTCTGCTTCTGTCAGGG-3′; and for YB-1 was 5′-CGGCGGGGGGGGCGGGG-3, which were cloned separately into the px458 vector (Addgene, Cambridge, MA, USA). Plasmid construction, transfection into sensitive and FR-resistant CRC CICs using Lipofectamine 2000 from Invitrogen (Carlsbad, CA, USA), and isolation of clonal CICs were conducted as described previously [172]. To evaluate the gene editing activity of gRNA, the genomic DNAs of gRNA-transfected CICs were extracted, and the CD44v6 and YB-1 genes were amplified using sequence-specific primers followed by digestion with T7 endonuclease 1 (T7E1) from New England Biolabs (Ipswich, MA, USA) at 37 °C for 30 min. The digested products were analyzed with agarose gel electrophoresis. Subsequently, the cells were cultured in the serum-free medium with basic fibroblast growth factor (bFGF, 10 ng/mL; R&D Systems) and epidermal growth factor (EGF, 10 ng/mL; R&D Systems), 5 μg/mL of insulin (Beyotime, Shanghai, China), and 2% of B-27 (Sigma, St. Louis, MO, USA) at 37 °C in a humidified atmosphere with 5% CO_2_ for 2 days. Single colonies were selected by antibiotic selection, passaged, and genotyped. The knockout mutants were confirmed by DNA sequencing and Western blot with CD44v6 and YB-1-specific antibodies.

### 4.7. RNA Silencing

For determining shRNA sequences used in this study, (1) coding nucleotide sequences of the genes were obtained from the NCBI, National Institutes of Health, website (www.ncbi.nlm.nih.gov); (2) hairpin shRNAs were designed to target a transcript sequence using the Broad Institute GPP Web Portal (http://portals.broadinstitute.org/gpp/public/); (3) sequences for cloning in pSico/pSicoR vectors were designed following the MIT Jackson Lab website (http://web.mit.edu/jacks-lab/protocols). The resulting pSicoR-CD44v6 shRNA1 (CD44v6 sh1), pSicoR-CD44v6 shRNA2 (CD44v6 sh2) transfectants constitutively silence respective CD44v6 genes in the cells. The pSicoR-Non targeted shRNA (NT sh) transfectants were used as control to the above shRNA transfectants (see Table 1 for shRNA sequences used in this study).

To rescue the shRNA knocked out gene, cells were transfected with the gene replacement vector containing the modified target gene (Knock-In (KI; shRNA-immune cDNA)) that no longer contains target sites for the shRNAs but still encodes a functional protein. This can often be achieved by utilizing one or more silent third-codon point mutations within the targeted region. This construct restores full function and rescues any loss-of-function phenotype as used in our previous study [57,58].

### 4.8. CR1SPR/Cas9 Knockout Mutant Gene Rescue Plasmids

When a gene is knocked out in cells, it is important to know if rescue of the original gene would reverse the downstream changes in order to authenticate the gene function. In both scenarios, reintroducing a mutant(s) and/or rescuing of a wildtype gene would fail because Cas9-gRNA by nature disrupts introduced genes. To overcome both hurdles, we used modified cDNAs for gene rescue. In case of CR1SPR/Cas9 gene knockout, because Cas9 cleaves where gRNA binds, one or a few nucleotides in the gRNAS binding site of the CD44v6 and YB-1 were modified by three nucleotides, while the amino acid sequences remained unchanged as described previously in generating shRNA resistant constructs and gRNA rescue constructs [57,58,173]. Total cell lysates were examined by Western blot analysis for the indicated proteins and β-tubulin or β-actin were used as internal standards. In some cases, total mRNAs were analyzed for the indicated mRNAs by QPCR.

### 4.9. Cell Growth Survival or Apoptosis Assays

Five thousand cells were plated in triplicate into 96-well plates containing appropriate growth media and incubated overnight. After 16 h of growth, cultures were incubated in media containing no serum for 16 h at 37 °C in 5% CO_2_, 95% air. Vehicle or chemotherapy drug was added to the plate. In each experiment, a total of five plates (six wells/treatment) were used. Experiments were repeated three times. The growth of these cells was determined by measuring increases in readings of ATP levels for viability (Cell Titer-Glo, Promega). The luminescent signal is proportional to cell viability and is measured using a luminometer (PerkinElmer). The Caspase-Glo^®^ 3/7 assay depends on the formation of free amino luciferin after adding caspase-3/7 DEVD-amino luciferin substrate to cell lysates and measuring amino luciferin by the luciferase present in the substrate reagent. The luminescent signal is proportional to caspase 3/7 activity and measured using a luminometer.

### 4.10. Tumor Sphere Formation

An optimized serum substitute (1 × B27 supplement) (from Creative Bio array) was freshly added to tumor formation medium (500 mL Dulbecco’s Modified Eagle Medium/F12 containing, 20 ng/mL epidermal growth factor; 10 ng/mL basic fibroblast growth factor; 5 μg/mL insulin; 0.4% bovine serum albumin). After harvesting the cells, 200 live cells in 200 µL of tumor sphere medium were suspended in ice. This suspension was kept on ice and mixed well for plating. PBS was added to the first and last columns (columns 1 and 12) of the 96-well plate to help minimize medium evaporation. This leaves 10 wells available for each row. Then, 200 μL aliquots of the cells were suspended in tumor sphere medium into each well (200 cells per well). For each treatment, cells were seeded into the wells of two rows for a total of 20 wells. The upper and lower edges of the 96-well plate were sealed with laboratory tape to avoid evaporation of medium, and cells were placed in an incubator at 37 °C and cultured in 5% CO_2_ for 10–14 days. After stipulated time of incubation, tumor sphere numbers were counted under a phase-contrast microscope using the 40× magnification lens. Data are presented as a percentage of wells containing tumor spheres compared to the total number of wells.

### 4.11. Cell Lysis and Immunoblotting

Cells were cultured until they were 75% confluent. They were washed twice at 4 °C with phosphate buffered saline (PBS), harvested with 0.05% Versene, and then washed in cold PBS again. The cells were pelleted by centrifugation at 5000× *g* for 2 min at 4 °C. The pellets were treated with the lysis buffer containing 1% Nonidet P-40, 0.5 mM EGTA, 5 mM sodium orthovanadate, 10% (v/v) glycerol, 100 µg/mL phenylmethylsulphonyl fluoride, 1 µg/mL leupeptin, 1 µg/mL pepstatin A, 1 µg/mL aprotinin, and 50 mM HEPES, pH 7.5. The lysates were clarified by centrifugation at 12,000× *g* for 10 min at 4 °C and then stored at −80 °C as described previously [57,58,174,175,176]. Cell lysates (normalized for protein concentration) were analyzed by immunoblotting as described previously [57,58,174,175,176]. The proteins on the blots were analyzed with antibodies from commercial sources for specific antibodies for each experiment using appropriate primary antibodies (β-tubulin and β-actin were used as internal standards). Proteins probed with primary antibodies were detected by treatment with horseradish peroxidase-linked anti-rabbit or anti-mouse antibodies as secondary antibodies followed by treatment with luminol reagent (Santa Cruz Biotechnology). Each protein was analyzed in samples from at least three independent experiments from each set of tumor cells and from CICs.

### 4.12. Coimmunoprecipitation and Pulldown

The roles of CD44v6 knockout on colocalization of CD44v6, YB-1, and MDR1 in nuclear extracts were determined by pull down with YB-1 antibody followed by Western blotting analysis. After immunoprecipitation, cells were washed with ice cold PBS and lysed in lysis buffer as described above for 30 min. The 10% cell lysates were kept without antibody immunoprecipitation (IP) for input control, and were analyzed by SDS PAGE on the same gel with the Co-IP samples. Immunoprecipitation was done on cleared lysates (12,000 rpm for 15 min at 4 °C) with indicated antibodies and protein G agarose beads (Merck) at 4 °C overnight. The precipitates were washed (3 X) in lysis buffer and boiled in SDS-sample buffer containing 100 mM dithiothreitol (DTT). Whole cell lysates as well as immunoprecipitates were subjected to Western blotting analysis using CD44v6, MDR1, and YB-1 antibodies and were detected as above.

### 4.13. Plasmids and Reporter Assays

Expression vectors: pDSET YBX-1 pDESTmycYBX1 was a gift from Thomas Tuschl (Addgene plasmid # 19878; http://n2t.net/addgene:19878; RRID:Addgene_19878); pcDNA3-cmyc was a gift from Wafik El-Deiry Addgene plasmid # 16011; http://n2t.net/addgene:16011; RRID:Addgene_16011); pTK-TWIST was a gift from Bob Weinberg (Addgene plasmid # 36977; http://n2t.net/addgene:36977; RRID:Addgene_36977); pGEM-OCT4 was a gift from James Thomson (Addgene plasmid # 16352; http://n2t.net/addgene:16352; RRID:Addgene_16352); SOX2 (Origene) CD44v6 specific PCR amplification products were isolated with polyadenylated RNA from the HT29 cell line. The PCR product was cloned in pcDNA3.1 vector and used as previously described [58].

#### 4.13.1. Reporter Vectors

The MDR1 and CD44v6 reporter constructs were synthesized by Bio basic (US) and cloned into the firefly pGL3-basic vector (Promega, Madison, WI, USA) upstream of the Luciferase reporter gene. The constructs were named as follows: (1) mdr1 (1) contains the basal promoter (−2300/+1); (2) mdr1 (2) contains promoter site (−500/+1). Both of the MDR1 promoter constructs contain multiple YB-1 binding sites. For the CD44v6 promoter, the constructs were named as follows: (1) CD44v6 (1) contains promoter site (−2000/+1); (2) CD44v6 (2) contains promoter site (−700/+1). Both of the CD44v6 promoter constructs contain multiple YB-1 binding sites. The M50 Super 8x TOPFlash vector (plasmid 12456) with a luciferase gene under the control of seven TCF/LEF-binding sites, and the corresponding M51 Super 8x FOPFlash vector (plasmid 12457) with mutated TCF/LEF-binding sites were obtained from Addgene (Cambridge, MA, USA). The normalization vector pRL-TK Renilla with a HSV-TK promotor driving Renilla luciferase was purchased from Promega (Madison, WI, USA).

#### 4.13.2. Transient Transfection and Luciferase Reporter Assay

For the transient assays, 1.0 × 10^5^ cells from both cell lines were cotransfected using Lipofectamine LTX 2000 (Invitrogen, (Carlsbad, CA, USA)) with 1 μg of each Luciferase construct and 100 ng of pRL-SV40 vector (Promega), according to the manufacturers’ instructions. Firefly and Renilla Luciferase activities were measured in cell lysates 48 h after transfection using the DualGlo Luciferase Assay System (Promega, Madison, WI, USA) on a Veritas TM Microplate Luminometer (Perkin Elmer, Waltham, MA, USA) following the manufacturer’s protocol. All experiments were done in triplicate. Ratios of Renilla luciferase readings to firefly luciferase readings were taken for each experiment, and triplicates were averaged. The average values of the tested constructs were normalized to the activity of the empty pGL3-basic vector, which was arbitrarily set at value 1.

#### 4.13.3. β-Catenin/TCF Reporter Assays

All reporter gene assays were done in 96-well plates. Sensitive and resistant SW948 and HT29 cells (1.0 × 10^4^/well) were transfected with SuperTOPFLASH reporter (25 ng) and TK-Renilla (5 ng), and with the respective plasmid DNA as indicated using Lipofectamine™ 3000 transfection reagent (Invitrogen, (Carlsbad, CA, USA) according to the manufacturer’s protocol. Each transfection was adjusted to 150 ng DNA/transfection with pcDNA3.1 empty vector. Where indicated, cells were transfected at 50–70% confluency with shRNA constructs using Lipofectamine™ 3000 transfection Reagent in 6 cm petri dishes according to the manufacturer’s protocol 24 h before seeding the cells for the reporter assays. Then, 50 ng/mL of WNT3A was added 24 h after DNA transfection. Cells were lysed 72 h after DNA transfection with 1 × Passive Lysis Buffer (Promega), and the luciferase activity was measured using the Luminescence counter (PerkinElmer, Waltham, MA, USA). TOPFLASH experiments were normalized to cotransfected Renilla gene expression.

### 4.14. Primer Design and PCR

- RNA extraction and cDNA synthesis [177]

Harvested cells were transferred to 1.5 mL Eppendorf tubes with a small amount of 1x PBS, and 1 mL of TRIzol (Invitrogen, Carlsbad, CA, USA) was added. After vortexing, the tube was kept in room temperature for 5 min. Then, 200 μL chloroform was added and left at room temperature for 10 min. The tube was centrifuged at 1300 rpm for 15 min at 4 °C. The upper phase containing RNA was transferred to a new tube, and 600 μL ice cold isopropanol was added. The tube was inverted several times, kept in ambient temperature for 10 min, and centrifuged at 1200 rpm for 13 min at 4 °C. After removing the supernatant, the white RNA pellet was washed with 1 mL of 75% alcohol and left air-dried. Then, the sample was dissolved in 50 μL DEPC-treated water. The quality and quantity of extracted RNA was checked by a spectrophotometer. The extract was electrophoresed on 1% agarose gel. DNA contamination was removed from all RNA samples by treating the samples with DNAase. Then, 500 ng of RNA was used for cDNA synthesis. In total, 1 μL primer, 1 μL buffer (5 x), 0.5 μL RNase inhibitor, 1 μL dNTP (10 mM), and 0.5 μL Reverse Transcriptase (Thermo Fisher scientific) were mixed in a microtube (0.2 mL). The synthesis was performed at 50 °C for 60 min in a thermal cycler (BioRad).

- Primer design and semiquantitative RT-PCR [177]

Primers were designed by online Primer Quest Tool (https://eu.idtdna.com). The quality of designed primers was analyzed by OligoAnalyzer Tool software. The semiquantitative PCR primer sequences used for CD44 exon specific PCR are given in Supplemental Table S1, and the primer sequences used in analyses of various genes are discussed in Section 4.15. Semiquantitative PCR was done using different amounts of cDNA of RNA samples. In total, 1 μL forward (F) and reverse (R) primers were used. For each sample, PCR was repeated three times. Each reaction contained 1 μL of a cDNA sample, 0.5 μL of a primer, 5 μL Taq DNA Polymerase 2× Master Mix Red (Amplicon Co., Brighton, UK), and 3 μL of water in a final volume of 10 μL. Before the main reactions, the PCR conditions, including thermal conditions, the number of cycles, and the cDNA concentrations, were optimized. During the main PCR cycles, temperature conditions, including one initial denaturation cycle (3 min at 95 °C), was followed by 35 cycles with a denaturation step for 5 s at 95 °C and a combined annealing and extension step for 35 s at 61 °C. The PCR products were electrophoresed on 2.5% agarose, stained with ethidium bromide, and photographed. The analysis of band intensities was done by ImageJ software.

### 4.15. Quantitative Real-Time RT-PCR (QPCR)

Total RNA was isolated from cells after various treatments and transfections as described in the figure legends for each specified experiment using the RNeasy mini kit (Qiagen, Beverly, MA, USA) according to the standard protocol provided by the manufacturer, with on-column DNA digestion. RNA integrity and concentration were analyzed using Bioanalyzer, and 100 ng of RNA was retrotranscribed into cDNA using the First Strand cDNA synthesis kit from Roche Applied Science. SYBR Green technology (Bio-Rad, Thermo Fisher, Waltham, MA, USA) was used for all real-time PCR experiments. Amplification was done with the real-time PCR analyzer (Bio-Rad cfx96). The PCR mixture (25 µL) contained 12.5 µL of 2 SYBR Green PCR Master Mix (Bio-Rad), 5 µL of diluted RT product (1:20), and 0.5 µM sense and antisense primer sets. The real-time PCR assays were done in three individual experiments with duplicate samples using standard conditions in a CFX96 real-time PCR detection machine. After incubations at 95 °C for 3 min, the amplification protocol consisted of 50 cycles of denaturing at 95 °C for 10 s, annealing, and extension at 60 °C for 30 s. The standard curve was made from a series dilution of template cDNA. Expression levels of tested genes were calculated after normalization with the housekeeping gene *GAPDH*. Primer sequences used in QPCR are presented in Table 2.

### 4.16. Chromatin Immunoprecipitation (ChIP) Assay

The chromatin immunoprecipitation (ChIP) assay was done using the ChIP assay kit (Upstate Biotechnology) following the manufacturer’s directions as described [58]. After crosslinking with formaldehyde, nuclear fractions from SW948-FR/SQ/CICs were immunoprecipitated with 5 μg of anti-CD44v6 or anti-YB-1 antibody, or with 1 μg of normal mouse IgG for 3 h. Chromosomal DNAs were purified and analyzed using semiquantitative PCR to detect the MDR1 and the CD44v6 promoter regions (Figure 6D for MDR1, and Figure 6I for CD44v6 promoters). Nuclear CD44v6, or YB-1-associated chromatins from v6 Mu1, YB-1 Mu3, and Vector transfected SW948-FR CICs were immunoprecipitated with YB-1 or CD44v6 antibodies for 3 h. Chromosomal DNAs were purified and analyzed using QPCR with primers for YB-1 sites of MDR1 (Figure 6F) to detect the MDR1 promoter regions. Similarly, nuclear CD44v6, or YB-1-associated chromatins from V6 Mu1, YB-1 Mu3, and Vector transfected SW948-FR CICs were immunoprecipitated with YB-1 or CD44v6 antibodies for 3 h. Chromosomal DNAs were purified and analyzed using QPCR with primers for YB-1 sites of CD44v6 (Figure 6K) to detect the CD44v6 promoter regions. Control IgGs were used as negative controls for immunoprecipitation. Chromatin inputs were used as loading controls for PCR. The primers used for ChIP PCR studies are presented in Supplemental Table S3.

### 4.17. In Vivo Tumorigenic Potential of CICs

All animal studies described were approved by the IACUC protocol **(**# IACUC-2019-00829 approved till 09/24/2022) from the Medical University of South Carolina and conducted in accordance with the National Institutes of Health Guide for the Care and Use of Animals. The nonobese diabetic/severe combined immunodeficient (NOD/SCID) female mice weighing ≈25 g and aged ≈5–6 weeks were obtained from the Jackson Laboratory and was used for the in vivo experiments. v6-Mu1 and YB-1 Mu3 knockout SW948-FR CICs were collected at 5 × 10^5^ cells/mL in physiological saline. Matrigel (Becton, Dickinson) was added to the cell suspension at a ratio of 1:2, and 200 μL of the cell suspension was subcutaneously injected into mice to induce tumor growth. The tumor sizes were measured by a caliper every 5 days, and tumor volume was calculated as (length (mm) × width (mm) × width (mm))/2. Sixty days later, the mice were sacrificed, and the solid tumors were collected. The tumor sizes and weights were examined to evaluate the tumor development (Figure 7A,B). Total RNAs extracted from the fresh tumors were processed for QPCR analysis for Cyclin D1, BCL2, FZD1, GINS1, and MMP9 (Figure 7D). Tumor lysates were analyzed for CD44v6, YB-1 and MDR1 by Western blot analyses (Figure 7C).

### 4.18. Statistics

A two-tailed Student’s *t*-test was used to compare mean values between sensitive and resistant cells using the following parameters: mean ΔΔCT values for QPCR; mean colony number for soft agar growth assays; mean densitometry values for QPCR and WB; mean percentage of cell viability assay (Cell Titer-Glo) and FACS analysis; mean luminescence for ATP activity in cell growth, Caspase Glow assays in apoptosis measurements; mean tumor weights in xenograft studies. Chi-squared analysis was performed to compare incidences between sensitive and resistant cells for the following assays: number of positive wells containing tumor spheres in the sphere formation assay; numbers of mice developing tumors in xenograft studies. For experiments involving three or more groups, statistical significance was calculated with GraphPad Prism Software v.8 (San Diego, CA, USA) using a 1-way or 2-way ANOVA with a Bonferroni’s posttest, Student’s *t* test, or log-rank (Mantel-Cox) test where appropriate (GraphPad Prism Software v.8 (San Diego, CA, USA)). Data are represented as the mean ± SD.

## 5. Conclusions

**Figure 8:**
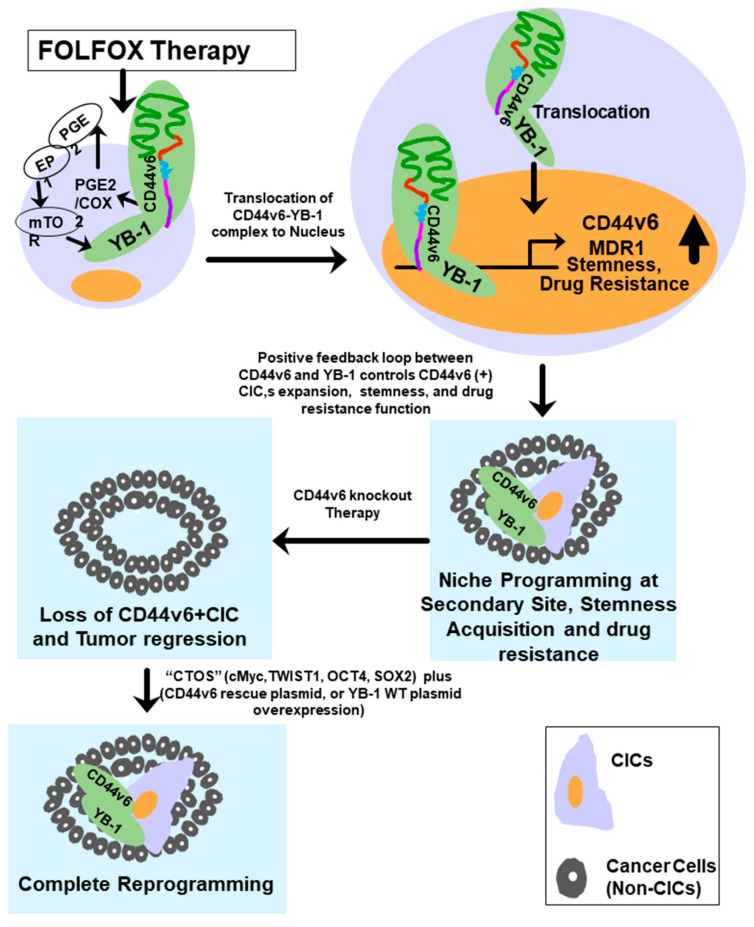
Proposed model for a positive feedback loop coupling YB-1 activation and CD44 alternate splicing and CD44v6 then sustains cancer initiating cell proliferation and stemness.

In conclusion, collectively, our data (Figure 8) indicate that (1) FOLFOX therapy induces overexpression of CD44v6, and of YB-1, a key oncogenic transcription factor to maintain stemness of CRC CICs; (2) our data link CD44v6-induced PGE2 with mTOR signaling to induce YB-1 expression; (3) we identified a novel function for CD44v6 in transcriptional modulation through nuclear translocation of CD44v6 and complex formation with stemness-associated transcription factors, including YB-1 in CICs, where YB-1 exerted its specific functions in cancer initiating cells via CD44v6 and MDR1 DNA transcription. As a result, a positive feedback loop couples YB-1 activation and CD44 alternative splicing to sustain CD44v6 and FOLFOX resistance through MDR1 expression; (4) in addition, we demonstrated that YB-1 is associated with CD44v6 in a nuclear complex. In CD44v6 Mu1 SW948-FR CICs, ectopic expression of the YB-1, and CD44v6-rescue plasmids enhanced expression of proliferation/anti-apoptosis/invasion/stemness associated Cyclin D1, BCL2, FZD1, GINS-1, and MMP9 genes providing evidence that CD44v6-elicited expressions of stemness related genes are mediated through CD44v6 and YB-1([178], (Supplemental Table S2)); (5) CD44v6-YB-1 signaling has an essential role in the activation and reversion of the pluripotency of differentiated cancer cells; (6) owing to their potential roles in chemoresistance, targeting CD44v6 significantly reduces tumor formation in vitro and in vivo. Thus, CD44v6 signaling could be a potential therapeutic target molecule combined with conventional chemotherapy to elevate the chemosensitivity of CRC (Figure 8).

## Figures and Tables

**Table 1:** shRNA sequence in pSico and pSicoR vectors (https://jacks-lab.mit.edu/protocols).

Genes	Primers	
	Sense Sequence (5′–3′)	Antisense Sequence (5′–3′)
CD44v6 shRNA1	TCCTCCCAGTATGACACATATTTTCAAGAGA -AATATGTGTCATACTGGGAGGTTTTTTC	TCGAGAAAAAACCTCCCAGTATGACACATATT-TCTCTTGAA-AATATGTGTCATACTGGGAGGA
CD44 shRNA2	TGGACCAATTACCATAACTATTTTCAAGAGA AATAGTTATGGTAATTGGTCCTTTTTTC	TCGAGAAAAAAGGACCAATTACCATAACTATT TCTCTTGAA-AATAGTTATGGTAATTGGTCCA

**Table 2:** Real-time PCR (QPCR) primers for various genes used in this study.

Genes	Primers	
	Forward Sequence (5′–3′)	Reverse Sequence (5′–3′)
NFkB	GTGACAGGAGACGTGAAGATG	TGAAGGTGGATGATTGCTAAGT
E2F1	TCCCTGAGCTGTTCTTCTG	CCTCCCTCACTTTCCCAATAAA
STAT3	GAGAAGGACATCAGCGGTAAG	CAGTGGAGACACCAGGATATTG
RUNX2	CGGAATGCCTCTGCTGTTAT	TGTGAAGACGGTTATGGTCAAG
Snail	ACTATGCCGCGCTCTTTC	GCTGGAAGGTAAACTCTGGATTA
TWIST1	AGACTCTGGAGCTGGATAACT	GCCTGTCTCGCTTTCTCTTT
SOX2	GGACTGAGAGAAAGAAGAGGAGAG	CGCCGCCGATGATTGTTATTA
Cyclin D1	GGTTCAACCCACAGCTACTT	CAGCGCTATTTCCTACACCTATT
FZD1	AAGACCGAGTGGTGTGTAATG	TGGCCATGCTGAAGAAGTAG
GINS1	TCAGGTGGACGAAGTGATTTG	CGAAGCAAGCGGTCATACA
MMP9	GAACTTTGACAGCGACAAGAAG	CGGCACTGAGGAATGATCTAA
Lgr5	GGGAAACGCTCTGACATACA	CTTCTGTGGGTACGTGTCTTAG
OCT4	GGAGGAAGCTGACAACAATGA	CTCTCACTCGGTTCTCGATACT
c-Myc	AAGCTGAGGCACACAAAGA	GCTTGGACAGGTTAGGAGTAAA
EpCAM	AGCTGGTGTTATTGCTGTTATTG	GCATCTCACCCATCTCCTTTAT
ALDH1	CTTGGAATTTCCCGTTGGTTATG	GAGAGCAGTGAGAGGAGTTTG
Nanog	GCCTGTAGTCCCAGCTATTTG	GGAGTGCAGTGGTGTGATATT
ZEB1	GGCTCCTATAGCTCACACATAAG	TGCTGAAAGAGACGGTGAAG
CD44v6	GACAGAATCCCTGCTACCAATAG	TCCTTCGTGTGTGGGTAATG

## Data Availability

The datasets generated during the current study are not publicly available because the data are used in the manuscript and in funding opportunity.

## Supplementary Materials

The following are available online at https://www.mdpi.com/1422-0067/22/2/753/s1.

## Abbreviations

CD44s	Standard isoform of CD44 having no variant exons
CD44v	CD44 splice variant
CD44v6	variant 6 of CD44
v6	CD44v6
sh	shRNA
v6 Mu1	v6 knockout Mu1
v6 Mu2	v6 knockout Mu2
YB-1 Mu3	YB-1 knockout Mu3
YB-1 Mu4	YB-1 knockout Mu4
CIC	Cancer initiating cell
CRC	Colorectal cancer
5-FU	5-Fluorouracil
OXA	Oxaliplatin
FOLFOX	5-FU pls OXA plus leucovorin
ALDH1	Aldehyde dehydrogenase 1
MDR1	Multidrug-resistance protein 1
IP	Immunoprecipitation
YB-1	Y-box binding protein-1
WB	Western blotting
SQ	Subcutaneous
CTOS	c-Myc, TWIST1, OCT4, and SOX2
TF	Transcription Factors
